# Nephronophthisis-Associated *CEP164* Regulates Cell Cycle Progression, Apoptosis and Epithelial-to-Mesenchymal Transition

**DOI:** 10.1371/journal.pgen.1004594

**Published:** 2014-10-23

**Authors:** Gisela G. Slaats, Amiya K. Ghosh, Lucas L. Falke, Stéphanie Le Corre, Indra A. Shaltiel, Glenn van de Hoek, Timothy D. Klasson, Marijn F. Stokman, Ive Logister, Marianne C. Verhaar, Roel Goldschmeding, Tri Q. Nguyen, Iain A. Drummond, Friedhelm Hildebrandt, Rachel H. Giles

**Affiliations:** 1 Department of Nephrology and Hypertension, University Medical Center Utrecht, Utrecht, The Netherlands; 2 Division of Geriatrics & Palliative Medicine, Department of Internal Medicine, University of Michigan, Ann Arbor, Michigan, United States of America; 3 Department of Pathology, University Medical Center Utrecht, Utrecht, The Netherlands; 4 Nephrology Division, Massachusetts General Hospital and Department of Genetics, Harvard Medical School, Charlestown, Massachusetts, United States of America; 5 Department of Medical Oncology, University Medical Center Utrecht, Utrecht, The Netherlands; 6 Department of Medical Genetics, University Medical Center Utrecht, Utrecht, The Netherlands; 7 Division of Nephrology, Boston Children's Hospital, Massachusetts, United States of America; 8 Howard Hughes Medical Institute, Chevy Chase, Maryland, United States of America; Washington University School of Medicine, United States of America

## Abstract

We recently reported that centrosomal protein 164 (CEP164) regulates both cilia and the DNA damage response in the autosomal recessive polycystic kidney disease nephronophthisis. Here we examine the functional role of *CEP164* in nephronophthisis-related ciliopathies and concomitant fibrosis. Live cell imaging of RPE-FUCCI (fluorescent, ubiquitination-based cell cycle indicator) cells after siRNA knockdown of *CEP164* revealed an overall quicker cell cycle than control cells, although early S-phase was significantly longer. Follow-up FACS experiments with renal IMCD3 cells confirm that *Cep164* siRNA knockdown promotes cells to accumulate in S-phase. We demonstrate that this effect can be rescued by human wild-type *CEP164*, but not disease-associated mutants. siRNA of *CEP164* revealed a proliferation defect over time, as measured by CyQuant assays. The discrepancy between accelerated cell cycle and inhibited overall proliferation could be explained by induction of apoptosis and epithelial-to-mesenchymal transition. Reduction of *CEP164* levels induces apoptosis in immunofluorescence, FACS and RT-QPCR experiments. Furthermore, knockdown of *Cep164* or overexpression of dominant negative mutant allele *CEP164 Q525X* induces epithelial-to-mesenchymal transition, and concomitant upregulation of genes associated with fibrosis. Zebrafish injected with *cep164* morpholinos likewise manifest developmental abnormalities, impaired DNA damage signaling, apoptosis and a pro-fibrotic response *in vivo*. This study reveals a novel role for *CEP164* in the pathogenesis of nephronophthisis, in which mutations cause ciliary defects coupled with DNA damage induced replicative stress, cell death, and epithelial-to-mesenchymal transition, and suggests that these events drive the characteristic fibrosis observed in nephronophthisis kidneys.

## Author Summary

Nephronophthisis is a leading inherited cause of renal failure in children and young adults. This work contributes to understanding of the disease mechanism of nephronophthisis, which is characterized by multi-cystic and fibrotic kidneys. The genes mutated in patients with nephronophthisis all seem to encode proteins involved in cilia function, and some of them are recently reported to also function in DNA damage signaling. We investigated how loss of cilia and impaired DNA damage signaling could cause the excessive fibrosis seen in nephronophthisis. Studies during the past decade have focused on treating the cysts of this early-onset renal disease. However, we think that understanding and curing the fibrosis seen in these patients will provide new treatment opportunities. Our work gives insight into the orchestration of downstream effects on the cellular level after loss of nephronophthisis gene *CEP164* as a result of loss of cilia and accumulating DNA damage signaling.

## Introduction

Nephronophthisis (NPHP) is an autosomal recessive polycystic kidney disease (PKD) attributed to dysfunction of the primary cilia [1], antennae-like structures projecting from the cell surface which have sensory or mechanical functions [2]. To date, mutations in seventeen genes have been identified as causing NPHP, yet fewer than half of all NPHP cases segregate with these disease loci [3]. Although ciliary dysfunction with consequent defective planar cell polarity among the epithelial cells in the kidney is believed to be the fundamental etiology of cystogenesis in both NPHP and other types of PKD [4], the overall size of kidneys in NPHP is considerably smaller than in autosomal dominant PKD [5]. This discrepancy is partly due to tubulointerstitial renal fibrosis in NPHP, which is far more evident than in autosomal dominant PKD-affected kidneys. Epithelial-to-mesenchymal transition (EMT) is a hallmark of tubulointerstitial renal fibrosis [6]. Recent studies associating NPHP proteins with defective DNA damage response (DDR) signaling [7], [8] support the notion that accumulation of DNA damage and cilia loss result in cell cycle arrest or cell death with associated renal function loss and fibrosis [9], but exactly how these processes are linked remains unknown.

One of the proteins linking these cellular processes in NPHP is centrosomal protein 164 (*CEP164*) (NM_014956, NP_055771). CEP164 regulates primary cilium formation [10] by promoting vesicular trafficking to the mother centriole during initiation of ciliogenesis [11]. Germline mutations in *CEP164* have been reported in families with *NPHP15* (MIM:614845) [7]. Furthermore, *CEP164* has a role in DDR signaling [7], [12], [13]. Cep164 interacts with checkpoint kinases ATR and ATRIP *in vivo* [12] and localizes with DNA damage proteins TIP60, SC-35 and phosphorylated Chk1 [7]. *CEP164* expression is cell cycle stage-dependent; most protein is present at the end of S phase and the beginning of the G_2_/M phase when cilia are not typically present. Reduction of endogenous levels of *CEP164* by siRNA knockdown in HeLa cells abrogates the G_2_/M checkpoint [12], suggesting a critical role in cell cycle regulation. Because disturbance of the cell cycle contributes to the cystic and fibrotic renal phenotype of NPHP [14], we interrogated whether these non-ciliary functions of *CEP164* might contribute to the particular phenotype observed in NPHP kidneys.

Here we investigate the role of *CEP164* in the cell cycle, particularly in S-phase progression and proliferation. We test wild-type and mutant alleles of *CEP164* and verify that disease alleles of *CEP164* affect cilia as well as cell cycle progression. Live cell imaging studies suggest that CEP164 protects cells from apoptosis. Furthermore we observe upregulation of EMT and fibrosis markers as a result of reduced cellular levels of *CEP164 in vitro* and *in vivo* that could partially explain the cystic and fibrotic renal phenotype of *CEP164* mutant patients.

## Results

### *CEP164* knockdown accelerates cell cycle, but delays S phase progression

**Figure 1:**
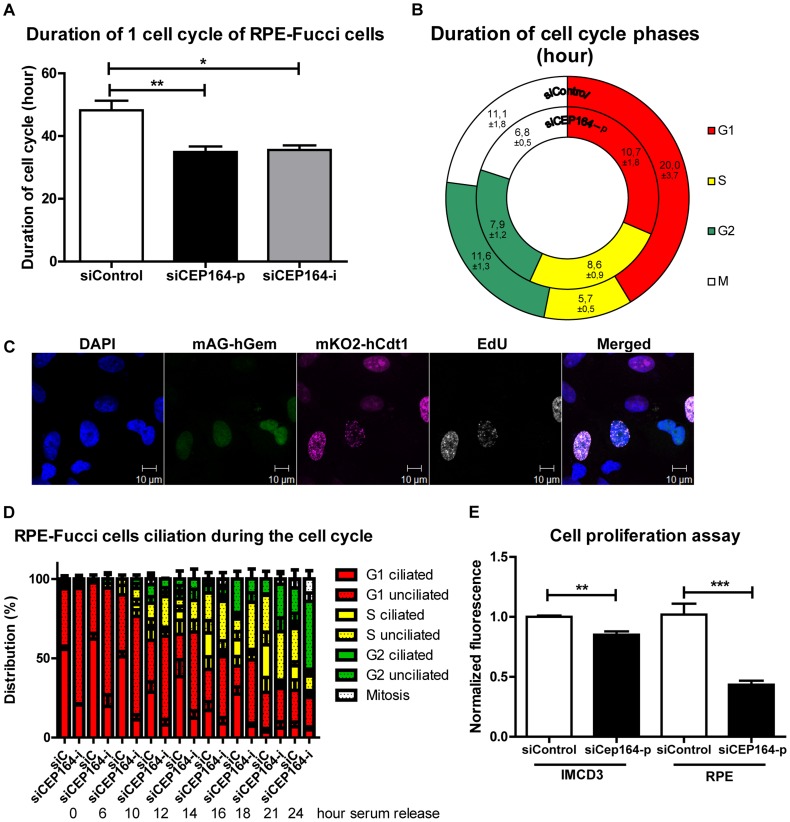
CEP164 regulates cell cycle progression and proliferation. (A) RPE-FUCCI cells and their daughter cells were tracked by live cell imaging for 72 hours (hr) after transfection. *CEP164* depleted cells have a quicker cell cycle (∼35 hr) compared to control cells (∼48 hr) (>25 cells and their daughter cells per position (n = 3) per experimental condition per experiment (n = 3), One-way ANOVA (Dunnett's post hoc) (*p<0.05). Error bars represent SEM. (B) Each cell cycle stage in siControl and si*CEP164* transfected cells was measured. S-phase took significantly longer in si*CEP164* transfected cells and their daughter cells (8.6 hour) compared to control (5.7 hour) (**p<0.01). G_1_-phase is significantly shorter in si*CEP164* transfected cells (10.7 hour) compared to control (20 hr) (*p<0.05). G_2_- and M-phase were almost significantly shorter in si*CEP164* transfected cells (7.9 and 6.8 hr respectively) compared to control (11.6 and 11.1 hour respectively; both p = 0.06) (>25 cells and their daughter cells per position (n = 3) per experimental condition per experiment (n = 3), ± represent SEM, see Figure S1E for details. (C) Fluorescence images of RPE-FUCCI cells expressing mKO2-hCdt1 (30/120) (magenta) and mAG-hGem (1/110) (green) constructs were pulsed for 30 minutes with EdU (10 µM) and stained with Alexa anti-EdU-647 to visualize cells in early S-phase (white) and DAPI to visualize the nuclei (blue). Cells expressing both constructs also show EdU incorporation (white). Scale bar represents 10 µm. (D) Quantification of a time series of serum released RPE-FUCCI cells after 24 hour serum starvation. Cell cycle stage and ciliation were quantified and correlated. Both decreased ciliary frequency and increased cell cycle entry were observed in *CEP164* depleted cells. Error bars represent SEM. (E) Quantification of RPE and IMCD3 cell proliferation using the CyQUANT NF Cell Proliferation Assay Kit. Fluorescence intensities of quadruplicate samples after 72 hours after transfection were measured. A significant reduction in cell number is visible after respectively *CEP164* and *Cep164* knockdown (n = 3, **p = 0.007, ***p = 0.001). Error bars represent SEM.

To establish the cell cycle progression of cells after knockdown of endogenous *CEP164*, we generated RPE-FUCCI cells [15] stably expressing mKO2-hCdt1(30/120) (red) and mAG-hGem(1/110) (green) [16] and confirmed that knockdown due to either a pool of four siRNAs (siCEP164-p) or an individual siRNA (siCEP164-i) causes down-regulation of *CEP164* mRNA levels (Figure S1A), resulting in a 5-fold reduction of cilia in these cells capable of forming cilia after serum starvation (Figure S1B–D). Live cell imaging of unsynchronized RPE-FUCCI cells for 72 hours (Figure S1E and Movies S1–2) reveals a significantly shorter cell cycle in si*CEP164* transfected cells than control non-targeting siRNA (siControl) transfected RPE-FUCCI cells (∼35 hours versus ∼48 hours) (Figure 1A). However, these same videos show that si*CEP164* transfected cells remain significantly longer in early S-phase (8.6 hours) compared to siControl transfected cells (5.7 hours). Accordingly, G1, G2 and M phases in siCEP164 transfected RPE-FUCCI cells are shorter (Figure 1B, S1E and Movies S1–2). RPE-FUCCI cells expressing both mKO2-hCdt1(30/120) and mAG-hGem(1/110), always demonstrated EdU incorporation, supporting the accuracy of the early S-phase values scored (Figure 1C). To determine how defective ciliogenesis in the siCEP164 cells is affecting cell cycle progression, we performed a time series experiment with RPE-FUCCI cells synchronized at G_0_/G_1_. The reduced ciliation frequency observed in cells with reduced levels of CEP164 (Figure S1B–C) is consistent with the increased tendency to enter the cell cycle and with the speed with which they proceed through the cell cycle. Both decreased ciliary frequency and increased cell cycle entry were observed. It takes siControl transfected cells about 10 hours to enter S-phase, whereas siCEP164 treated cells require only 6 hours (Figure 1D).

### Despite accelerated cell cycle, proliferation is decreased after *CEP164* knockdown in IMCD3 as well as RPE cells

We next wanted to validate whether the accelerated cell cycling of si*CEP164* knockdown cells conferred a growth disadvantage as had been suggested by Chaki *et al.* [7]. We performed CyQUANT NF Cell proliferation assays and then measured fluorescence 72 hours after siRNA knockdown of *CEP164* or siControl in RPE and IMCD3 cells. Mouse Cep164 siRNA reduces endogenous mouse *Cep164* expression significantly (Figure S2A–B). DNA staining by the CyQuant assay revealed a decreased cell number after knockdown in both cell lines (**p<0.01; Figure 1E). Standard growth curves in IMCD3 cells reveal a significant growth advantage for cells treated with siCtrl over cells treated with siCep164. Previously, these results were seen to be rescued by WT-CEP164 in IMCD3 cells [7]. These results are supporting the conclusion that increased cell cycle progression does not result in decreased population doubling time in the context of CEP164 loss.

### Wild-type CEP164 overexpression rescues S phase accumulation

**Figure 2:**
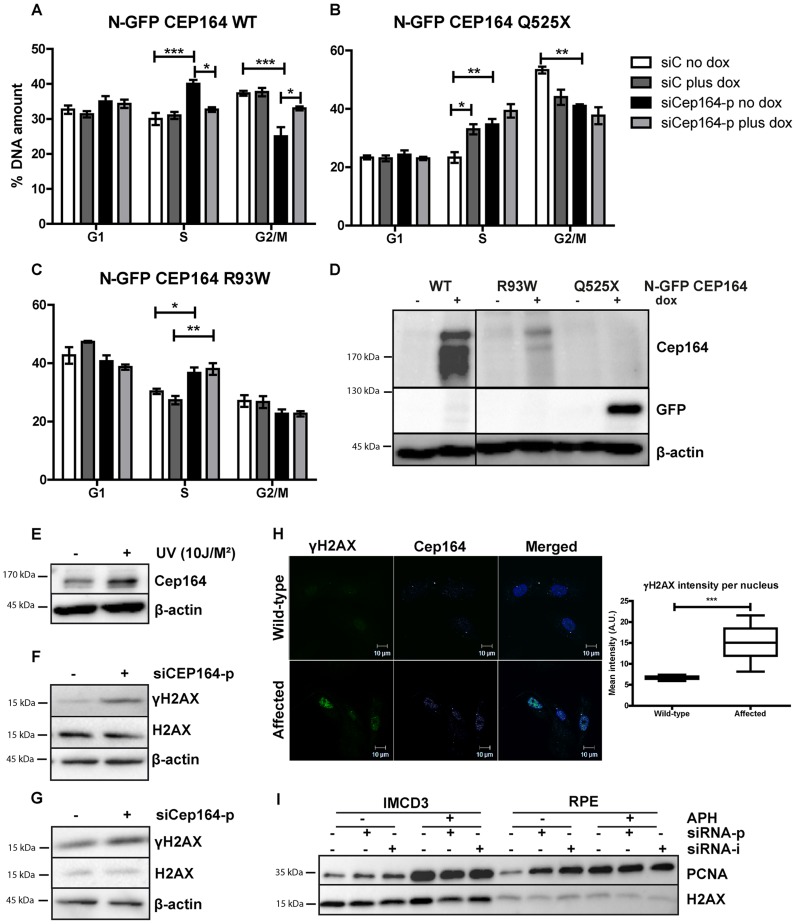
Knockdown of *Cep164* causes S-phase delay. (A–D) Endogenous *Cep164* knockdown leads to block in S-phase and is rescued by inducible human wild type *CEP164* but not by human mutant *CEP164*. Under thymidine synchronization, *Cep164* knockdown cells (black) were lagging in the transition from S to G_2_/M phase in comparison to the control siRNA treated cells (white) in all IMCD3 *N-GFP-CEP164-WT* (A) IMCD3 *N-GFP-CEP164-Q525X* (B) and IMCD3 *N-GFP-CEP164-R93W* (C) cell lines. Upon doxycycline induction of wild type human CEP164 construct IMCD3 *N-GFP-CEP164-WT* (light grey) cells were rescued from arrest in S-phase (A). In contrast, overexpression of the cDNA clone that represents the human truncating mutant *N-GFP-CEP164-Q525X* cells did not rescue the S-phase lag. In addition, overexpression of *N-GFP-CEP164-Q525X* (dark grey) caused an increase of cells in S-phase, indicating a dominant negative effect of the human truncating mutant (B). Cells were not rescued by *N-GFP-CEP164-R93W* (C) from the S-phase lag upon doxycycline induction. P values were calculated using two-way ANOVA and Bonferroni multiple comparison test. (n = 3, *p<0.05). (D) Western blot showing expression of N-GFP CEP164 WT and R93W by CEP164 antibody and N-GFP CEP164 Q525X by GFP antibody upon doxycycline induction. β-actin is used as loading control. (E) Lysates of IMCD3 cells were made 1 hour after 10J UV exposure. Western blot was performed for Cep164 and loading control β-actin. Stabilization of Cep164 is visible after DNA damage induction (n = 3). (F) Lysates of RPE cells were made 56 hours after knockdown of *CEP164* with 20 nM siRNA. Upregulation of DNA damage marker γH2AX is shown on western blot with loading controls H2AX and β-actin (n = 3). (G) Lysates of thymidine synchronized IMCD3 cells were made 56 hours after knockdown of *Cep164* with 20 nM siRNA. Upregulation of DNA damage marker γH2AX is shown on western blot with loading control H2AX and β-actin 6 hours after release of the thymidine block (n = 3). (H) Urine derived renal cells stained for γH2AX (green) and CEP164 (magenta) of NPHP patient and unaffected control. γH2AX intensities were quantified by ZEN2011 software. T-test reveals statistical difference (***p<0.001) (n = 15). (I) S-phase marker PCNA expression is increased in RPE and IMCD3 cells transfected with siCep164/CEP164p and –i compared to control transfected cells. β-actin was used as loading control. APH exposure (18 hour, 400 nM) enhanced the PCNA expression levels in both cell lines.

We stably transfected murine renal inner medullary collecting duct (IMCD3) cells and RPE cells with doxycycline (Dox) -inducible constructs expressing GFP-tagged CEP164 wild-type (Figure 2D, S2D, S5A) or human disease-associated cDNA N-GFP-CEP164-, -R93W and -Q525X. Upon induction with doxycycline, all constructs showed GFP expression at the base of the cilium, in the cytoplasm and occasionally in the nucleus (Figure S2D, S5A). These cells were then transfected with either siControl or siCep164-p/-i or siCEP164-p/-i to reduce endogenous expression (Figure S2A,B). We observed a reduction in the percent of ciliated cells in all cell lines after knockdown of endogenous *Cep164/CEP164* (Figure S2E, S5B). Rescue of cilia numbers in cells was observed upon induction of wild-type allele CEP164-WT (Figure S2E, S5B) by the addition of doxycyline, a finding which is consistent with our previously published results and extends upon them, although not quantified in a 3D experimental setting in this study [7]. To induce cell cycle arrest and attain synchronization, we used a double thymidine block prior to release (Figure S2C) and then followed the IMCD3 inducible stable cell lines using FACS. Upon siRNA knockdown of *Cep164*, cell cycle histograms of IMCD3-N-GFP-CEP164-WT cells revealed increased accumulation of DNA in S-phase at the expense of G_2_/M phase (40%±2) when compared to the control siRNA treated cells (30%±3) (Figure 2A) indicating cell cycle delay or arrest in transition from S to G_2_/M phase. Upon doxycycline induction of wild-type human CEP164 construct N-GFP-CEP164-WT cells were rescued from S-phase arrest (32.7%±1.2) (Figure 2A). The observed S-phase block cannot be rescued by overexpression of the human nonsense mutant CEP164-Q525X, a mutation from NPHP family F59 [7] (Figure 2B); however, it should be noted that simply expressing the CEP164-Q525X allele alone had a nearly identical statistically significant effect as siCep164 treatment, suggesting a dominant negative interference of N-GFP-CEP164-Q525X with murine endogenous Cep164 function (Figure 2B). To rule out the possibility of clonal drift, we repeated these experiments in IMCD3 polyclonal lines expressing N-GFP-CEP164-WT and N-GFP-CEP164-Q525X (Figures S2F) and again observed a rescue with the wild-type allele and a dominant negative effect upon expression of the Q525X allele.

Cells expressing NPHP missense mutation N-GFP-CEP164-R93W [7] exhibited S-phase accumulation upon siRNA knockdown of endogenous mouse *Cep164* (increase from 30% to 36.7%±3.2) indicating that this disease-causing variant affects this function of CEP164 (Figure 2C). We conclude that CEP164 plays a role in early S-phase progression and that mutations in *CEP164* associated with NPHP are defective in this function. Because S-phase arrest may reflect increased DNA damage response signaling, we examined Cep164 levels in the presence or absence of DNA damage and observed increased levels of Cep164 protein (Figure 2E). Importantly, IMCD3 and RPE cells depleted of *Cep164* accumulate the DNA damage marker phosphorylated H2AX (γH2AX) (Figure 2F–G), which is accompanied by stabilization of PCNA, after transfection with a species-specific pooled (siCEP164-p/siCep164-p) or individual siRNA (siCEP164-i/siCep164-i) or exposure to replication stress agent aphidicolin (APH) (Figure 2I). To examine the pathophysiological relevance of these data, we obtained a urine sample from a newly diagnosed and untransplanted NPHP patient and isolated urine-derived renal epithelial cells. Compared to a healthy age- and gender-matched control, localization of CEP164 was observed to be more nuclear and γH2AX was quantitatively more evident (***p<0.001) (Figure 2H). Although this is an isolated patient, these data would indicate that DDR processes are relevant to advent of NPHP.

### Apoptosis and DNA damage are enhanced in *CEP164* depleted cell populations

**Figure 3:**
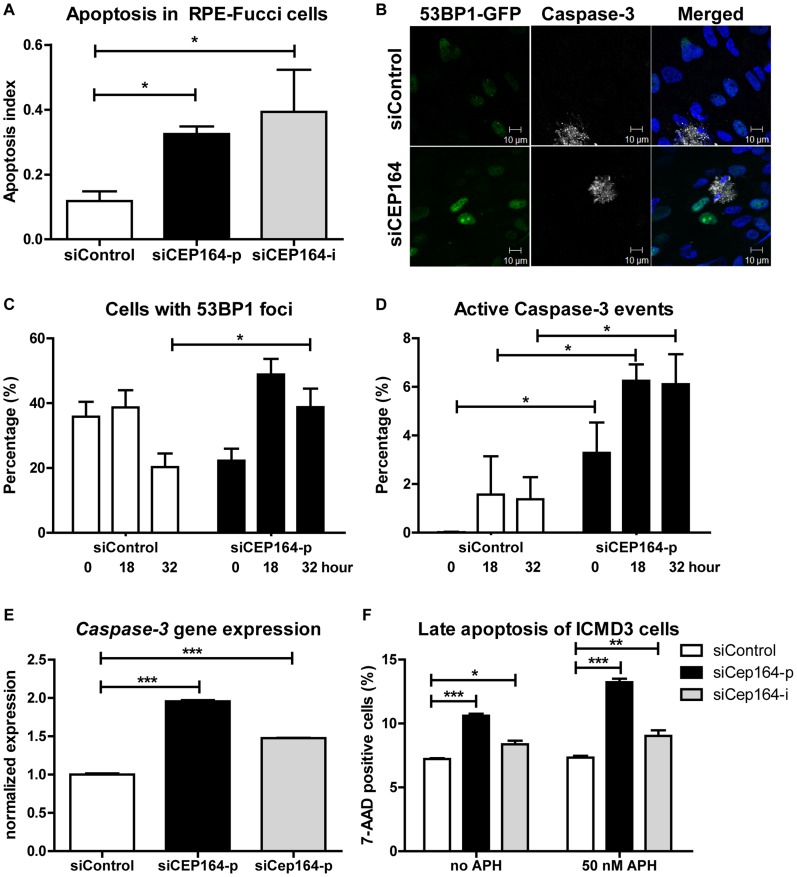
Induced apoptosis and DNA damage accumulation after loss of *CEP164*. (A) RPE-FUCCI cells and, after mitosis, their daughter cells are followed during 72 hours after transfection. The number of apoptotic events was scored and normalized to the total number of RPE-FUCCI cells (150 cells). *CEP164* depleted cells show more apoptosis in their population compared to control cells (n = 3, One-way ANOVA (Dunnett's post hoc) *p<0.05). Error bars represent SEM. (B–D) RPE 53BP1-GFP cells were transfected with siControl or si*CEP164* oligos and serum starved for 24 hours. Samples were fixed after 0, 18, and 32 hours and prepared for confocal imaging (B). (C) Cells with 53BP1-GFP foci were quantified and normalized against the total number of cells. At t = 0, and 18 hours cells displayed no significant differences in the number of scored 53BP1 foci after knockdown of *CEP164*, but at t = 32 (*p<0.05) more 53BP1 foci are observed. (D) More apoptosis events (*p<0.05) are seen at all time points after knockdown of *CEP164*. (n = 3,>150 events scored per condition, error bars represent SEM.). (E) Relative gene expression levels of pro-apoptotic marker Caspase-3 as measured by RT-QPCR in RPE-FUCCI and IMCD3 cells, normalized to *RPLP0* and *Rpl27* respectively. Total RNA was isolated 24 hours after transfection with siControl or si*CEP164* oligos. After 24 hours of transient transfection Caspase-3 mRNA levels are increased (***p<0.0001; n = 3). Error bars represent SEM. (F) IMCD3 cells were transfected with control or *Cep164–p/i* siRNA. Cells were incubated with 0 or 50 nM aphidicolin (APH) to induce replicative stress for 16 hours. Cells were harvested and stained for 7-AAD to measure late apoptosis (n = 3, 10,000 events, error bars represent SEM). P-values were calculated using two-way ANOVA and Bonferroni multiple comparison tests.

Despite the fact that reduction of cellular levels of *CEP164* by si*CEP164* knockdown results in a quicker cell cycle than controls (Figure 1A), we consistently observed a decreased cell number during CyQUANT assays using different cell lines (Figure 1E). This paradox led us to investigate whether apoptosis might explain the discrepancy between the accelerated cell cycle in RPE-FUCCI cells after knockdown of *CEP164* and decreased net proliferation which was determined by several assays. The time-lapse data from RPE-FUCCI cells transfected with si*CEP164* or control were analyzed for the number of cells characteristically appearing apoptotic (passive or Brownian movement, blebbing, detaching, and/or lacking fluorescence) within 72 hours of filming. These events were significantly higher after *CEP164* knockdown, normalizing for the total number of cells per field (*p<0.05) (Figure 3A). For molecular analysis of apoptotic markers, RNA from RPE-FUCCI cells and IMCD3 cells was isolated after transfection with control or si*CEP164-p or siCep164-p* oligos respectively and we performed RT-QPCR to measure *Caspase-3* mRNA expression (Figure 3E). Twenty-four to 48 hours after transfection there was increased expression of *Caspase-3* mRNA (***p<0.0001) which we visualized by live cell imaging of dual immunofluorescent staining of Annexin V and Caspase-3 substrate (Figure S5C–D). As further validation, we transfected *CEP164* siRNA into RPE cells stably expressing 53BP1-GFP, a protein which accumulates at double strand breaks [17], and stained those cells for Caspase-3 after fixation. RPE nuclei showing 53BP1-GFP foci were counted as well as Caspase-3 positive nuclei and were normalized against the total number of nuclei analyzed. After 32 hours more 53BP1-GFP positive cells were counted in si*CEP164* cells compared to controls (*p<0.05) (Figure 3B–C). During all time points significantly (*p<0.05) more apoptosis events were scored (Figure 3B,D). We performed a FACS assay for measuring apoptosis of *Cep164* siRNA transfected IMCD3 cells incubated with 50 nM aphidicolin (APH) for 16 hours, a treatment which causes replicative stress and synchronizes cells in S-phase [8]. *Cep164* knockdown caused apoptosis of IMCD3 cells which was further enhanced by APH treatment (Figure 3F), suggesting that S-phase prolongation and/or replicative stress may predispose renal cells to apoptosis (Figure 2).

### *CEP164* regulates epithelial-to-mesenchymal transition

**Figure 4:**
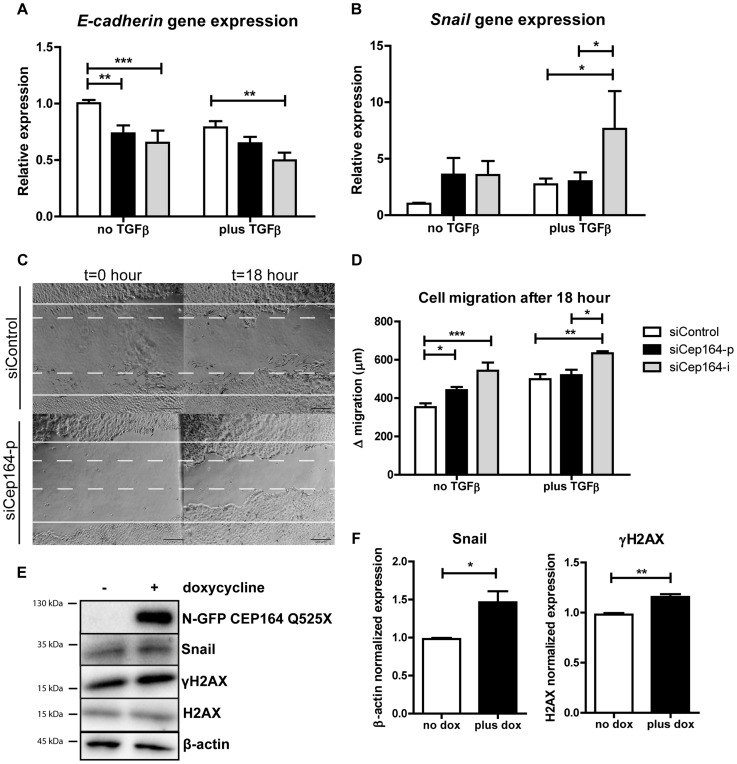
Loss of Cep164 induces EMT. (A) Relative gene expression levels of epithelial marker E-cadherin as measured by RT-QPCR in IMCD3 cells, normalized to RPL27. Total RNA was isolated 6 days after transfection with siControl or siCep164 oligos with or without TGFβ incubation (5 ng/mL) in serum-free medium. After 6 days of transient transfection (siRNA transfection occurred at day 0 and day 3) E-cadherin mRNA levels are significantly decreased. (**p<0.01; n = 4, error bars represent SEM). (B) Relative gene expression levels of mesenchymal marker Snail as measured by RT-QPCR in IMCD3 cells, normalized to RPL27. Total RNA was isolated 6 days after transfection (siRNA transfection occurred at day 0 and day 3) with siControl or siCep164 oligos with or without TGFβ incubation (5 ng/mL) in serum-free medium. After 6 days of transient transfection Snail mRNA levels (*p<0.05) are increased. (n = 4, error bars represent SEM). (C) Representative scratch healing images of IMCD3 cells taken by light microscope before and 18 h after a scratch was made in a confluent monolayer of cells. The white solid lines represent the wound edges at t = 0 h and the white dashed lines indicate the edges at t = 18 h. Scale bar represents 200 µm. (D) Quantification of absolute distance (µm) of cell migration after 18 hours after a scratch. IMCD3 *Cep164* depleted cells migrate (*p<0.05) more than siControl cells 48 hour after transfection. TGFβ incubation (5 ng/mL) in serum-free medium enhances this effect (n = 4, error bars represent SEM). P-values were calculated using two-way ANOVA and Bonferroni multiple comparison test. (E–F) Lysates of IMCD3 *N-GFP-CEP164-Q525X* cells treated with doxycycline were made 24 hours after addition to the culture medium. Western blot was performed for Snail and γH2AX and loading controls β-actin and H2AX respectively. Upregulation of both Snail and γH2AX are visible after induction of dominant negative allele *N-GFP-CEP164-Q525X* (n = 3). Quantification of the protein expression of Snail (Student's t-test *p<0.05) and γH2AX (**p<0.01) in lysates of IMCD3 *N-GFP-CEP164-Q525X* cells normalized to loading control β-actin and H2AX respectively was performed using Image Lab software (n = 3, error bars represent SEM).

Epithelial-to-mesenchymal transition (EMT) slows down cell proliferation and provides cells an alternative to apoptosis [18]. A pro-fibrotic mesenchymal transition is characterized by expression of Snail [19]. E-cadherin expression decreases as cells lose their epithelial characteristics and become more mesenchymal [20]. We investigated the role of siCep164 (Figure S3F) in the induction of EMT using TGFβ1 incubation (5 ng/mL) as a positive control for EMT in IMCD3 cells [21], [22]. Six days after knockdown of *Cep164* in IMCD3 cells we measured decreased gene expression levels of *E-cadherin* (Fig. 4A) and increased levels of *Snail* (Fig. 4B). *Tieg1*, *TGF-β1*, α*SMA, Fibronectin1* and *CTGF* (Figure S3A–E) are concomitantly upregulated after Cep164 depletion as measured by RT-QPCR (*p<0.05).

Because mesenchymal cells migrate faster than epithelial cell populations [21], we performed a scratch wound migration assay [23] to investigate the cell migration capacity of IMCD3 cells after knockdown of *Cep164*. Cells with reduced levels of *Cep164* migrated significantly faster than siControl cells (*p<0.05). TGFβ1 incubation had a comparable effect on migration in this experimental set-up (Figure 4C–D). Accordingly, expressing the dominant negative allele *N-GFP-CEP164-Q525X* resulted in increased Snail and γH2AX protein levels (*p<0.05) (Fig. 4E–F).

Finally, we investigated the role of siCep164 in the induction of fibrosis in mouse embryonic fibroblasts (MEFs). Six days after knockdown of *Cep164* in MEFs (Figure S4F) we measured increased levels of *TGF-β1, Fibronectin1* and *CTGF*, but not of *Tieg1* and α*SMA* (Figure S4A–E), by RT-QPCR. We conclude that *Cep164* has a role in inducing EMT and fibrosis in renal epithelial and mesenchymal cells. Furthermore, our data suggest this effect to be possibly specific to the kidney; RPE cells in similar experimental settings do not undergo EMT and do not migrate (Figure S5E–G).

### Apoptosis, DNA damage and fibrosis are enhanced in *cep164* depleted zebrafish

**Figure 5:**
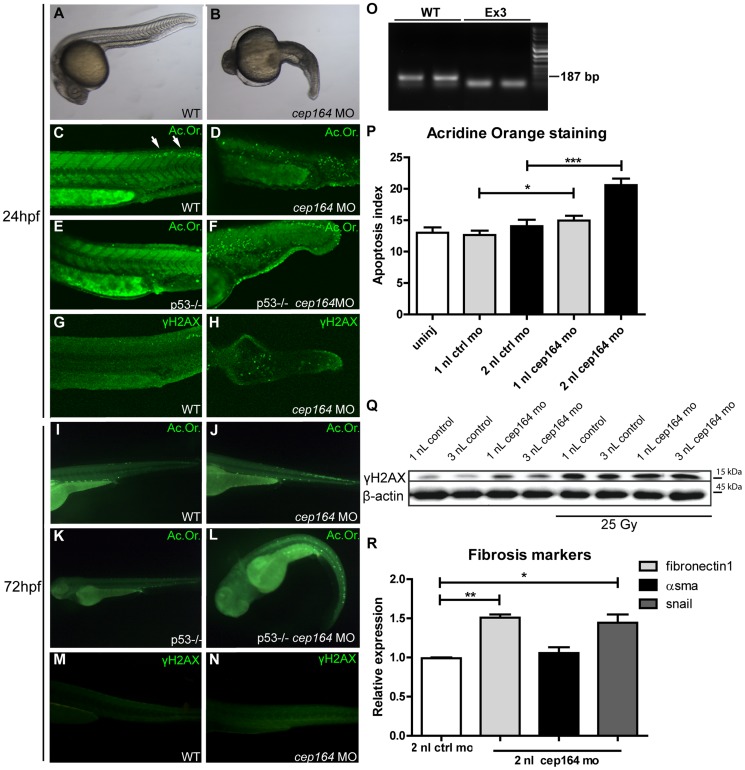
Apoptosis, DNA damage signaling and fibrosis in zebrafish. Zebrafish *cep164* knockdown leads to developmental abnormalities associated with apoptosis and DNA damage. A *cep164* morpholino targeting the exon 3 splice donor reduces wildtype mRNA (lanes WT) and results in a smaller cep164 RT-PCR product indicating an internal deletion (lanes Ex3) (O) was injected into 1–2 cell embryos. Compared to wild-type embryos at 24 hpf (A), morphant embryos (B) were stunted and displayed shorten body axis, body axis curvature, and edema. No cysts or other specific phenotypes were observed in the pronephros of morphant fish. Acridine orange staining revealed a low level of apoptosis in control injected WT (C) and *p53* -/- embryos (E) restricted primarily to the posterior neural tube (arrows in C). *cep164* knockdown induced widespread apoptosis (D) in the trunk and tail which was not affected by p53-deficiency as indicated by the *cep164* knockdown-induced apoptosis in *p53-/-* mutants (n = 30) (F). Staining with γH2AX antibody revealed enhanced DNA damage signaling in *cep164*-deficient embryos (n = 10) (H) but not controls (G). Acridine orange staining (Ac. Or.) also revealed a low level of apoptosis in 72 hours post fertilization (hpf) control injected WT (I) and *p53-/-* embryos (K). *cep164* knockdown induced widespread apoptosis (J) which was not affected by p53-deficiency as indicated by the *cep164* knockdown-induced apoptosis in *p53*-/- mutants (n = 45) (L). Quantified acridine orange staining (Ac. Or.) reveals significantly increased apoptosis in WT zebrafish after *cep164* knockdown (P). Student's t-test were used to calculate p-values (*<0.05, ***<0.001) (n = 70 in 4 experiments, error bars represent SEM). Staining with γH2AX antibody revealed enhanced DNA damage signaling in *cep164*-deficient embryos (N) but not control injected WT embryos (n = 10) (M). Western blot of γH2AX protein levels from lysates of 15 pooled embryos, two hours after irradiation with 25 Gy, show increased DNA damage signaling (Q). RT-QPCR reveals significant induction of *snail* and *fibronectin1* in cep164 MO injected embryos at 32 hpf (R). mRNA expression from 12 pooled embryos is normalized to 2 nL control MO injected zebrafish. Student's t-test was used to calculate p-values (*<0.05, **<0.01).

To evaluate *cep164* loss of function *in vivo* we performed morpholino oligonucleotide (MO) knockdown in zebrafish using a different MO targeting the splice donor site of exon 3 (Figure 5O) than previously published [7]. This more effective morpholino induced consistent and robust developmental abnormalities. *cep164* knockdown caused microcephaly, shortened body axis, axis curvature and edema (Figure 5B, L) compared to wildtype zebrafish after control injection (Figure 5A) and in general recapitulates the results of the other published MO [7]. This phenotype was associated with massive cell death as demonstrated by widespread acridine orange (Ac.Or. green) staining in morphant embryos (Figure 5D) compared to control injected siblings (Figure 5C). Homozygous *p53* mutant zebrafish embryos injected with *cep164* morpholino displayed cell death as well compared to control injections (Figure 5E–F). DDR signaling was also activated in morphant embryos as indicated by an increased signal of γH2AX immunofluorescence compared to control (Figure 5G–H). 72 (hours post fertilization, hpf) morphant embryos displayed increased apoptosis (Figure 5I–L, 5P) and increased γH2AX levels as well (Figure 5M–N–Q). No specific pronephros DNA damage or apoptosis accumulation was observed; however, the pronephros at this embryonic stage is exquisitely regenerative. Similarly, we examined *cep164* morphant zebrafish for induction of EMT and a profibrotic response after depletion of cep164. Indeed, RT-QPCR revealed significant induction of *snail* and *fibronectin1* in cep164 MO injected embryos at 32 (Figure 5R), 72 and 96 (Figure S6) hpf.

## Discussion

**Figure 6:**
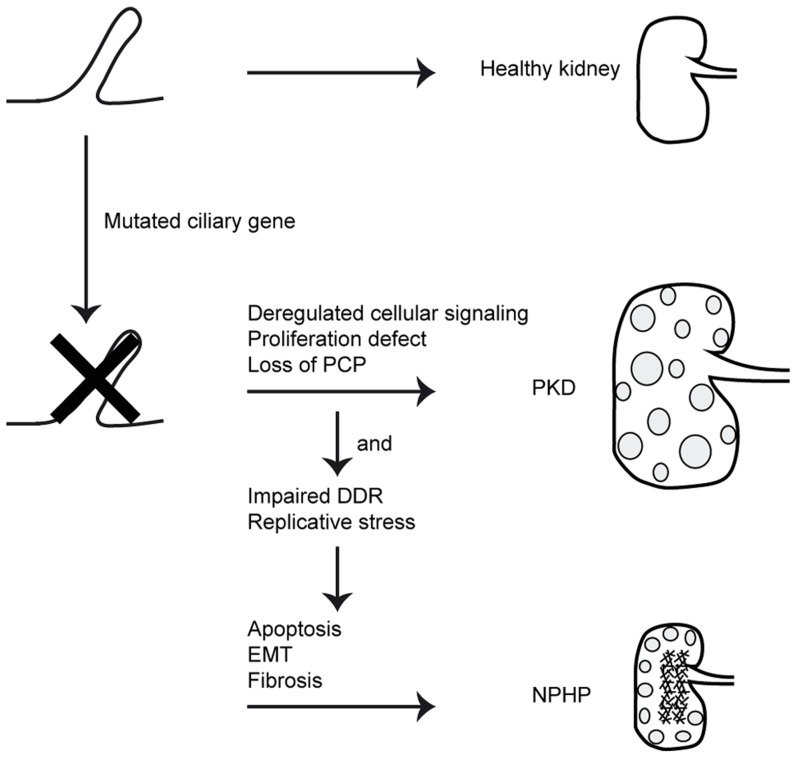
Schematic overview of signaling cascade involved in development of PKD and NPHP. Mutations in ciliary genes cause ciliary defects. Ciliary defects result in proliferation defects, loss of planar cell polarity (PCP) and deregulated cellular signaling. This causes cystic kidney disease. When the gene mutated is also involved in the DNA damage response (DDR) or DNA replication, impairments of these processes cause apoptosis, epithelial-to-mesenchymal transition (EMT) and consequently fibrosis in the NPHP patients.

NPHP is a common cause of renal end-stage disease in children and young adults. Although NPHP-associated ciliary defects and impaired DNA damage response have been associated with *CEP164* dysfunction (NPHP15) [7], [10], [12], [13], the exact mechanism linking these processes to NPHP is unclear. Here we identify novel functions for CEP164 relevant to NPHP pathogenesis, namely in cell cycle progression, apoptosis, EMT and fibrosis regulation. We show that despite accelerated cell cycle progression, total cell number is decreased after *CEP164* knockdown. Our data further indicate a role for CEP164 in S-phase progression. Accumulation of cells in S-phase could be rescued by wild-type *CEP164*, but not by its disease-associated variant alleles. We observed that *Cep164*-loss promotes apoptosis *in vitro* characterized by increased levels of 53BP1 and γH2AX. Zebrafish with reduced levels of cep164 show developmental abnormalities, increased apoptosis, enhanced DDR signaling and a profibrotic response, demonstrating the *in vivo* relevance of our findings. These novel functions are highly relevant to the etiology of NPHP which features increased apoptosis and fibrosis. Our data suggests functional similarity between CEP164 and at least one other NPHP protein, *GLIS2* (*NPHP7*), which also protects renal cells from apoptosis and fibrosis [5]. With two of the seventeen known nephronophthisis-associated proteins clearly associated with these processes, as well as the ongoing large-scale proteomics efforts to understand the nephronphthisis interactome (www.syscilia.org) [24], we anticipate that additional NPHP-genes will be implicated in these processes. In short, we propose that these non-ciliary functions of NPHP genes help to explain differences in disease progression between NPHP and other types of PKD (Figure 6).

Studies from Graser *et. al.* show that *CEP164* expression is cell cycle stage-dependent [10]. Most protein is present at the end of the S-phase and the beginning of G_2_/M in HeLa cells. Knockdown of *CEP164* in HeLa cells showed abrogation of the G2/M checkpoint [12], suggesting a role of *CEP164* in the G_2_/M checkpoint. In line with these published findings, we find that *CEP164* knockdown in RPE-FUCCI cells accelerates G_1_, G_2_ and M cell cycle phase durations but delays S-phase progression. Human wild-type *CEP164*, but not its disease-associated mutants, rescue IMCD3 cells from accumulation in S-phase. Activation of the intra-S checkpoint occurs when replication forks cannot stall at damaged DNA [25], ensuring that the cell cycle progression only reinitiates after damage repair. Because *CEP164* mutant alleles were not able to rescue S-phase accumulation, we suggest that replicative stress might contribute to NPHP development in general. In a similar manner, *Nek8(NPHP9)*-mediated replication stress contributes to the NPHP etiology [8].

EMT is a hallmark of tubulointerstitial renal fibrosis [6]. Upon *CEP164* mutation, induction of apoptosis might compete with EMT as has previously been described [18], [26], [27]. The reduced total cell number after si*Cep164* can partially be explained by induced apoptosis but probably by transdifferentiation of the epithelial kidney cells as well. Accordingly, mesenchymal marker Snail is increased upon reduction of cellular levels of Cep164 in conjunction with a decrease of epithelial marker E-cadherin, indicating that cells lose their epithelial characteristics. The pathological significance of the tubular EMT in renal fibrosis is becoming increasingly accepted [28]. *Snail* activation associates with patients' renal fibrosis, and disrupts renal homeostasis [29]. The principal effector cells of renal fibrosis are myofibroblasts; evidence suggests that both cells of epithelial origin and cells of mesenchymal origin as progeny for myofibroblasts [6]. We observe that epithelial cells *in vitro* and *in vivo* undergo the process of EMT upon *Cep164* knockdown, a proposed mechanism contributing to the rise of myofibroblasts during fibrosis. Furthermore, we saw that *Cep164* depletion was capable of inducing fibrotic genes in MEF cells, presumably because MEFs are already mesenchymal and thus no longer require EMT. These results obtained from cell types of both epithelial and mesenchymal origin indicate that *Cep164* loss can induce fibrosis regardless of the origin of the myofibroblast. In NPHP patients, excessive deposition of extracellular matrix (fibrosis) by mesenchymal cells replaces functional tissue [30].

Briefly, this manuscript shows *in vitro* S-phase arrest, quicker cell cycle progression and EMT and fibrosis induction upon loss of *Cep164*. Accumulation of DNA damage signaling during replicative stress could be the cause of the observed apoptosis. Apoptosis is known to contribute to initiation of renal cyst formation [31], [32]. Our data support the hypothesis proposed by Choi *et al.* which states that, in addition to cilia loss-of-function, replicative stress contributes to the disease mechanism of NPHP as well [8]. We show that loss of *Cep164* results in EMT and fibrosis in different cell types as well as in zebrafish. The induced overall pro-apoptotic and pro-fibrotic response of different cell types may explain the non-cystic features of nephronophthisis such as reduced kidney size (Figure 6). Since the fibrotic nature of NPHP kidneys is progressive with a time window of several years for therapeutic intervention, understanding and curing this aspect of juvenile kidney disease will potentially delay the need for renal replacement therapy. Our data support the hypothesis that the NPHP-interactome encoded by the 17 NPHP genes coordinates cilia loss-of-function with concomitant DNA damage response, apoptosis, and the creation of a pro-fibrotic environment, all of which directly contribute to the renal phenotype in these patients.

## Materials and Methods

### Ethics statement

Renal epithelial cells were obtained from a nephronophthisis patient that had been included in the AGORA (Aetiologic research into Genetic and Occupational/environmental Risk factors for Anomalies in children) biobank project. The regional Committee on Research involving Human Subjects (CMO Arnhem/Nijmegen) approved the study protocol. Written informed consent was obtained from the patient and the parents.

All zebrafish experiments were approved by the Animal Care Committee of the University Medical Center Utrecht in the Netherlands.

### Urine-derived renal epithelial cells

Renal epithelial cells were obtained from a nephronophthisis patient and a healthy gender- and age-matched control. The patient was determined to have isolated clinical diagnosis of NPHP. Urine-derived renal epithelial cells were derived as we have previously described [33].

### IMCD3 cell culture

Mouse Inner Medullar Collecting Duct (IMCD3) cells were cultured in Dulbecco's Modified Eagle's Medium (DMEM):F12 (1∶1) (GlutaMAX, Gibco), supplemented with 10% Fetal Calf Serum (FCS) and penicillin and streptomycin (1% P/S). Cells were incubated at 37°C in 5% carbon dioxide (CO_2_) to approximately 90% confluence. IMCD3 cells were stably transfected with *CEP164* constructs in a retroviral vector (pRetroX-Tight-Pur) for doxycyclin-inducible expression. Inducible overexpression was obtained of N terminally GFP-tagged human full-length *CEP164* isoform 1 (NGFP-CEP164-WT), or truncated *CEP164*, corresponding with the p.Q525X mutation, or non-functional *CEP164*, corresponding with the p.R93W mutation by addition of 2 ng/ml doxycycline [7].

### RPE cell culture

Human retinal pigment epithelial (RPE) cells were cultured in DMEM∶F12 (1∶1) (GlutaMAX, Gibco), supplemented with 10% FCS and 1% P/S. Cells were incubated at 37°C in 5% CO_2_ to approximately 90% confluence. RPE cells were transfected with lentiviral vectors containing mKO2-hCdt1(30/120) and mAG-hGem(1/110). Fluorescent, ubiquitination-based cell cycle indicator (FUCCI) [16] expressing stable transformants were generated [15]. RPE 53BP1-GFP cells are described in Janssen *et. al.* [17] RPE cells were stably transfected with *CEP164* constructs in a retroviral vector (pRetroX-Tight-Pur) for doxycyclin-inducible expression. Inducible overexpression was obtained of N terminally GFP-tagged human full-length *CEP164* isoform 1 (NGFP-CEP164-WT). RPE cells were serum starved>24 hour in experiments for cilia quantification.

### MEF cell culture

Mouse embryonic fibroblasts (MEFs) were cultured in DMEM (Gibco), supplemented with 10% FCS and 1% P/S. Cells were incubated at 37°C in 5% CO_2_ to approximately 90% confluence.

### Transfections

At least 6 hours after plating, cells were transfected with Lipofectamine RNAimax (Invitrogen, 13778-075), according to the supplier's protocol. Opti-MEM (Invitrogen, 31985-062) was used to dilute the ON-TARGETplus siRNA SMARTpools (Thermo Scientific Dharmacon) for Non-targeting pool UGGUUUACAUGUCGACUAA/UGGUUUACAUGUUGUGUGA/UGGUUUACAUGUUUUCUGA/UGGUUUACAUGUUUUCCUA (D-001810-10), human *CEP164* GAGUGAAGGUGUAUCGCUU/GAGAAGUGGCGCAAGUAUU/GGACCAUCCAUGUGACGAA/GAAGAGUGAACCUAAGAUU (L-020351-02), human *CEP164* GAGUGAAGGUGUAUCGCUU (J-020351-17), or mouse *Cep164* GGAGAGUGCAGGAGGGAGA/ACCCAGUGCAGGCAGGAAA/AGUCAGAGAUCCACGGACA/CCACAGAAAGAAAACGAGA (L-057068-01), mouse *Cep164* CCACAGAAAGAAAACGAGA (J-057068-09) to 20 nM.

### Real time imaging

RPE-FUCCI cells were seeded in 8 well Lab-Tek Chamber Slides (Thermo Scientific) without addition of serum. After 16 hours, the cells were transfected with Lipofectamine RNAimax. Seven hours after transfection, medium in the Lab-Tek Chamber Slides was replaced with Leibovitz's medium without phenol red (Gibco), supplemented with 6% FCS, 1% P/S and 1% Ultraglutamine. Real time imaging was performed using a Zeiss microscope using a 10× lens. Every 15 minutes images were made of the RPE-FUCCI cells in LED, GFP and dsRED channels for 72 hours. Three positions were imaged per experimental condition. Images were processed with the MetaMorph software. GraphPad Prism 5.0 was used to perform one-way ANOVA with Dunnett's post test.

### Immunofluorescence and confocal imaging

For immunostaining, IMCD3, RPE-FUCCI, urine derived renal epithelial cells or RPE 53BP1-GFP cells were grown on coverslips and fixed for 30 minutes in 4%PFA at the indicated time points, followed by a 15 minutes permeabilization step in 0.5%Triton-X100/1%BSA/PBS. Primary antibody incubations (mouse anti-acetylated tubulin (Sigma, T7451, dilution 1∶20000), rabbit anti-CEP164, Novus 45330002, 1∶500, mouse anti-phospho-Histone H2A.X (Ser139), clone JBW301, Millipore 05-636, 1∶500 or rabbit anti-active Caspase-3 (BD Pharmingen, 559565, dilution 1∶250) were performed overnight in 1% BSA/PBS. Goat anti-mouse/rabbit Alexa 647 secondary antibody (Invitrogen, dilution 1∶500) incubations were performed for 2.5 hours at RT. DAPI incubations were performed for 10 minutes at RT. Coverslips were mounted in Fluormount G (Cell Lab, Beckman Coulter). Confocal imaging was performed using Zeiss Confocal laser microscope and images were processed with the ZEN 2011 software. Approximately 250 events per condition were scored. GraphPad Prism 5.0 was used to perform statistical analysis. To observe centrosomal localization of N-GFP-CEP164-WT, clonally inducible IMCD3 cell lines doxycylcin (Dox)-inducibly expressing human *N-GFP-CEP164-WT* were treated by double thymidine block (2 mM). Cells were also induced with doxycycline (10 ng/mL) during the thymidine block to express *N-GFP-CEP164-WT*. Cells were stained with CEP164-SR antibody followed by anti-rabbit-alexa fluor 594 antibody for confocal imaging to observe colocalization with the induced *N-GFP-CEP164-WT*-expressing cells. For live cell imaging, RPE cells were seeded in Lab-Tek Chamber Slides with cells at 30% confluency. RPE cells were transfected and after 16 hours, wells were washed once with PBS and once with 1× Binding Buffer (NucView Dual Apoptosis Kit for Live Cells, Biotium, 30067). Cells were incubated 40 minutes at RT with NucView 488 Caspase-3 substrate (5 µM) and CF 594-Annexin V (1∶40) in 1× Binding Buffer. Cells were washed once with 1× Binding buffer and confocal imaging was performed using a Zeiss Confocal laser microscope and images were processed with the LSM500 software. GraphPad Prism 5.0 was used to perform two-tailed student t-tests.

### EdU staining

To examine cells in S phase, RPE-FUCCI cells were seeded on coverslips. After 48 hour EdU (Invitrogen, A10044) incorporation took place for 30 minutes using 10 µM in culture medium. The cells were fixed in 3% PFA and washed with PBS. Cells were shortly incubated with EdU staining buffer (100 mM Tris pH 8,5; 1 mM CuSO_4_). Then the cells were incubated with EdU staining buffer containing Alexa Fluor 647 azide (1∶1000) (Invitrogen, A10277) and ascorbic acid (0.1 M) (Merck) for 30 minutes at RT in the dark. The coverslips were washed twice with PBS and incubated with DAPI for 30 minutes at RT in the dark. The coverslips were mounted with Fluormount G (Cell Lab, Beckman Coulter) after washing them once with PBS. Confocal imaging was performed using Zeiss Confocal laser microscope and images were processed with the ZEN 2011 software.

### CyQUANT NF cell proliferation assay

RPE and IMCD3 cells were transfected in 96 well plates seeded with cells at 30% confluency. CyQUANT NF reagent (Invitrogen, C35006) was prepared according to the manufacturer's protocol. After 72 hour of incubation, 50 µl of CyQUANT NF Cell Proliferation Assay reagent was added to each well after aspiration of medium. After incubation for 30 minutes at 37°C, fluorescence was measured (excitation 485 nm, emission 538 nm) on a Fluoroskan Ascent FL apparatus (Thermo Scientific, 374-90441C) using Ascent Software version 2.6. Blanc measurement subtraction was performed and GraphPad Prism 5.0 was used to perform two-tailed student t-tests.

### Real-Time-Quantitative PCR (RT-QPCR)

Cells were lysed and total RNA was isolated (RNeasy Mini Kit, Qiagen, 74106) and measured (NanoDrop spectrophotometer ND-1000, Thermo Fischer Scientific Inc.). cDNA was synthesized from 500 ng RNA template using the iScript cDNA Synthesis Kit (Bio-Rad, 170-8891) according to the supplier's protocol. Dilutions were made for RT-QPCR analysis to determine mRNA expression levels which were normalized against a reference gene. The iQ SYBR Green Supermix (Bio-Rad, 170-8880) was used to multiply and measure the cDNA with a CFX96 Touch Real-Time PCR Detection System (Bio-Rad). All samples were run in triplicate in 20 µl reactions. The following PCR program was used: 95°C for 3 min, followed by 40 cycles of 10 s at 95°C, 30 s at the indicated annealing temperature and 30 s at 72°C, then 10 s at 95°C followed by a melt of the product from 65°C–95°C. The primer sequences (Sigma) used and concomitant annealing temperatures are: hCEP164 forward 5′-GGCAAAGCTGTCAACTTCTGG, hCEP164 reverse 5′-GAACTGGGGCTAATGAGGAAC, 61°C, mCep164 forward 5′-AGAGTGACAACCAGAGTGTCC, mCep164 reverse 5′-GGAGACTCCTCGTACTCAAAGTT, 61°C, hRPLP0 forward 5′-TGCACAATGGCAGCATCTAC, hRPLP0 reverse 5′-ATCCGTCTCCACAGACAAGG, 58°C, hCaspase-3 forward 5′-ACATGGCGTGTCATAAAATACC, hCaspase-3 reverse 5′-CACAAAGCGACTGGATGAAC, 60°C, mE-cadherin forward 5′-CAGTTCCGAGGTCTACACCTT, mE-cadherin reverse 5′-TGAATCGGGAGTCTTCCGAAAA, 66°C, mCaspase-3 forward 5′-GGCTTGCCAGAAGATACCGGT, mCaspase-3 reverse 5′-GCATAAATTCTAGCTTGTGCGCGT, 67°C, mSnail forward 5′- CACACGCTGCCTTGTGTCT, mSnail reverse 5′- GGTCAGCAAAAGCACGGTT, 66°C, hSnail forward 5′-TCGGAAGCCTAACTACAGCGA, hSnail reverse 5′-AGATGAGCATTGGCAGCGAG, 64°C, mRPL27 forward 5′-CGCCCTCCTTTCCTTTCTGC, mRPL27 reverse 5′-GGTGCCATCGTCAATGTTCTTC, 53°C, hVimentin forward 5′-GACAATGCGTCTCTGGCACGTCTT, hVimentin reverse 5′-TCCTCCGCCTCCTGCAGGTTCTT, 67°C, ZfαSMA forward 5′- CATGTACCCGGGCATTGCAGA,, ZfαSMA reverse 5′- GGAAGGTGGAGAGAGAGGCCA, zfSnail forward 5′-CTCCTGCCCACACTGTAACCG, zfSnail reverse 5′-CATGCGACTGAAGGTGCGAGA, zfFibronectin1 forward 5′-TCCCAGACATCACGGGCTACA, zfFibronectin1 reverse 5′-GCATGAGTTCTGTCCGGCCTT, zfβ-actin forward 5′-TCTGGATCTGGCTGGTCGTGA, zfβ-actin reverse 5′- CTCCTGCTCAAAGTCCAGGGC 63°C. Taqman assays were performed to measure mouse CTGF (Applied Biosystems, probe number Mm01546133_m1), Tieg1 (Mm00449812_m1), TGFβ1 (Mm01178820_m1), ACTA2 (αSMA, Mm00725412_s1), and Fn1 (Mm01256744_m1) gene expression levels. The following PCR program was used: 40 cycles of 15 s at 95°C, 60 s at 60°C. The ΔΔCT method was used for statistical analysis to determine gene expression levels.

### Immunoblotting

Protein lysates were prepared using RIPA lysis buffer. To correct for protein content BCA protein assay (Pierce) was performed. Western blots were performed for *Cep164*. β-actin was used as loading control in combination with Coomassie Blue staining. After blotting, the PVDF membranes were blocked in 5% dried skim milk in TBS with 0.5% Tween. And western blots were performed for γH2AX, PCNA and Snail. H2AX and β-actin were used as loading control in combination with Coomassie Blue staining. After dry blotting (iBlot Dry Blotting System, Invitrogen, IB3010-01), the nitrocellulose membranes were blocked in 5% BSA in TBS with 0.5% Tween. The primary antibodies (rabbit anti-Cep164, Novus 45330002, 1∶2000, rabbit anti-H2AX (pSer^139^), Calbiochem DR1017, 1∶1000, mouse anti-phospho-Histone H2A.X (Ser139), clone JBW301, Millipore 05-636, 1∶1000 (Specificity of the gamma H2AX antibodies was determined by pre-treatment with phosphatases), rabbit anti-Histone H2A.X, Millipore 070627, 1∶1000, rat anti-PCNA, Antibodies Online ABIN334654, 1∶1000, rabbit anti-Snai1, Santa Cruz sc-28199, 1∶400, and mouse anti-β-actin AC-15, Sigma A5441, 1∶15000, rabbit anti-GFP Abcam, 1∶1000) were incubated overnight at 4°C. The secondary swine anti rabbit, goat anti rat and rabbit anti mouse antibodies which are HRP conjugated (DAKO, dilution 1∶2000) were incubated for 1 hour at RT. The ECL Chemiluminescent Peroxidase Substrate kit (Sigma, CPS1120-1KT) was used for development. Scans of the blots were made with the BioRad ChemiDoc XRS+ device with Image Lab software 4.0. GraphPad Prism 5.0 was used to perform two-tailed student t-tests.

### Fluorescence-activated cell sorting (FACS)

To investigate S-phase progression, dox-inducible non-clonally and clonally selected mouse IMCD3 cells expressing wild type human *CEP164* cDNA construct *N-GFP-CEP164-WT* or mutant human *CEP164* construct *N-GFP-CEP164-Q525X* or *N-GFP-CEP164-R93W* were transfected with either negative control siRNA (50 nM) or anti-mouse *Cep164* siRNA (50 nM) using Polyplus transfection reagents. Cells were treated for double thymidine block (2 mM) from time point 24–42 to 50–68 hrs post transfection. Cells were then also induced with doxycycline (10 ng/ml) at 24 hrs post siRNA transfection for expression of human wild type construct *N-GFP-CEP164-WT* or human mutant constructs. Cells were released from second thymidine block for 6 hrs and fixed with 2% PFA and stained with PI/RNAse staining solution. Events were acquired in a FACSCalibur flow cytometer (BD Biosciences) for the cell cycle histogram Mean and SD of percent of DNA amount for different phases (triplicate samples) were calculated and plotted as histograms.

### Apoptosis FACS

To quantify apoptosis, IMCD3 cells were plated and transfected with siControl or siCep164. After 24 hours cells were exposed to 0 and 50 nM aphidicolin for 16 hours. Cells were harvested and washed once with 1% BSA-PBS. Cells were collected in FACS tubes in 200 µl 1% BSA-PBS containing Vybrant DyeCycle Violet Stain (Invitrogen, V35003, 1∶1000) to stain living and early apoptotic cells (7 minutes at 37°C) and 7-AAD viability stain (eBioscience, 00-6993, 1∶60) to stain late apoptotic cells (10 minutes on ice). Cells were measured (10.000 events) with a BD FACSCanto II flowcytometer and analyzed using BD FACSDiva Software [34]. GraphPad Prism 5.0 was used to perform two-way ANOVA with Bonferroni post hoc test.

### Migration assay

IMCD3 cells were transfected overnight with non-targeting siControl or siCep164 oligonucleotides in 24 well plates seeded with cells at 40% confluency. 48 hour later, when the cells were>85% confluent, a plastic disposable pipette tip was used to create a scratch wound in the cell monolayer. After washing the wells once with PBS, the cells were incubated with serum-free medium for 18 hours containing no or 5 ng/mL TGFβ (Peprotech, 100-21). Images of the same positions of the scratch were made with a light microscope (4× objective) after 0 and 18 hours. Migration of cells was measured with Image-Pro. GraphPad Prism 5.0 was used to perform two-way ANOVA with Bonferroni post hoc test.

### Zebrafish morpholino injections

Wild-type and p53-/- embryos (*tp53 M214K*) [35] at the 1–2 cell stage were injected with 1 or 2 nL of a 0.1 mM antisense morpholino oligonucleotide targeting *Cep164* exon 3 in pure water with 0.1% Phenol Red using a nanoject2000 microinjector (World Precision Instruments). The sequence of the exon 3 morpholino was: TGTGTTGTGGAGTGTGTGTTACCAT. The sequence of the standard control morpholino was: 5′-CCT CTTACCTCAGTTACAATTTATA-3′. Primers amplifying exon 3 (Cep164 ex 2–4 product length = 187) forward primer GGTGCTGGAGGAGGATTATG and reverse primer GTAGTAGACCTCGCCCGTCA were used. For western blot 15 embryos were pooled in 60 µL Triton X-100 lysis buffer. For RT-QPCR 5 embryos were pooled in 100 µL TRIzol reagent (Invitrogen, 15596-026) and RNA was isolated following standard procedures.

### Zebrafish acridine orange staining

24 and 72 (PTU treated) hpf live dechorionated embryos are incubated in a 2 mg/mL solution of acridine orange (Sigma) in PBS for 30 min at room temperature. Embryos are washed quickly in E3, then 5×5 minutes in E3 and visualized on a Zeiss LSM5 Pascal confocal microscope. No autofluorescence was detected in the regions analyzed. GraphPad Prism 5.0 was used to perform two-tailed student t-tests.

### Zebrafish γH2AX staining

Anti-phospho H2AX antibody was a kind gift from James Amatruda (University of Texas Southwestern Medical Center, Dallas, Texas 75390). Embryos were fixed in 4% PFA overnight at 4°C and stained with Anti-phospho H2AX (1∶1500), Alexa 488 goat-anti-rabbit (Invitrogen A11008) secondary antibody and visualized on a Zeiss LSM5 confocal microscope.

## Supporting Information

Figure S1 Validation of RPE-FUCCI cells and knockdown of human *CEP164*. (A) Relative *CEP164* gene expression levels as measured by RT-QPCR in RPE-FUCCI cells, normalized to *RPLP0*. Total RNA was isolated 48 hours after transfection with siControl or siCEP164-p or -i oligos. After 48 hour of transient transfection *CEP164* levels are significantly reduced (***p<0.001) (one-way ANOVA (Dunnett's post hoc)) (n = 3, error bars represent SEM). (B) Depletion of *CEP164* by siRNA causes a ciliary defect in RPE-FUCCI cells 55 hours after transfection, of which the last 30 hours are serum-starved (***p<0.0001). Nuclei and cilia were scored to generate ciliary frequencies. si*CEP164* transfected cells manifest lower cilia frequencies (8–20%) compared to control transfected RPE FUCCI cells (50%). 300 cells per condition were analyzed. Error bars represent SEM. (one-way ANOVA (Dunnett's post hoc)). (C) RPE-FUCCI cells have a primary cilium in G_1_- and S-phase of the cell cycle, but not during G_2_- or M-phase (see also Figure 1D). Cells were immunostained for acetylated tubulin (white) and DAPI stains nuclei (blue). Scale bar represents 10 µm. (D) Fluorescence images of RPE-FUCCI cells expressing mKO2-hCdt1(30/120) during G_1_/early S-phase and mAG-hGem(1/110) constructs during the complete S-phase/G_2_ phase of the cell cycle, as previously characterized [15]. Cells expressing both construct simultaneously appear yellow/orange, which we classify as early S-phase. Mitotic cells express neither of these constructs and are not fluorescent. Scale bar represents 5 µm. (E) RPE-FUCCI cells and their daughter cells after mitosis are followed during 72 hours after transfection. Duration of each cell cycle stage in siControl and si*CEP164* transfected cells was measured. S-phase took significantly longer in si*CEP164* transfected cells and their daughter cells compared to control (**p<0.01). G_1_ phase was significantly shorter in si*CEP164* transfected cells compared to control (*p<0.05). G_2_- (p = 0.06) and M- (p = 0.06) phase were almost significantly shorter in si*CEP164* transfected cells compared to control (>25 cells and their daughter cells per position (n = 3) per experimental condition per experiment (n = 3), error bars represent SEM).
(TIF)

Figure S2 Validation of IMCD3 cells expressing *N-GFP- CEP164* alleles and knockdown of mouse *Cep164*. (A) *Cep164* mRNA expression 48 hour after siRNA transfection normalized to *RPL27*. (*p<0.05) One-way ANOVA, Dunnett's multiple comparisons test (n = 3, error bars represent SEM). (B) Western blot of IMCD3 cells transfected with siCep164-p with β-actin as loading control to quantify protein levels after knockdown. (C) Clonally doxycycline (Dox)-inducible IMCD3 cell line expressing human *N-GFP-CEP164-WT* was treated with doxycycline (10 ng/mL) during the double thymidine block (2 mM). Cells were fixed and stained with CEP164-SR antibody to observe colocalization. Distinct centrosomal localization of N-GFP-CEP164-WT (green), in the presence or absence of thymidine was observed when costained with CEP164-SR antibody (red). Scale bars represent 25 µm. (D) Induction of *N-GFP-CEP164* wild-type allele in IMCD3 cells with doxycycline results in expression of GFP-tagged CEP164 at the base ot he cilium as shown by immunofluorescence. Centrosomes were stained with Pericentrin (magenta) and cilia with Acetylated Tubulin (white). (E) Functional testing of *CEP164* alleles shows rescue of ciliation after knockdown of mouse *Cep164* after induction of wild-type human *CEP164* (**p<0.01) but not mutants (Q525X and R93W). Ciliary frequency was quantified from IMCD3 cells with stable *CEP164* constructs transfected with siCtrl (white) or si*Cep164* (black) and normalized to siCtrl. Transient knockdown results in significant loss of ciliation (***p<0.001). (>250 cells scored per condition, error bars represent SEM.). P-values were calculated using two-way ANOVA and Bonferroni multiple comparison test. (F) Endogenous *Cep164* knockdown in a non-clonally selected IMCD3 cell line leads to a block in S-phase under thymidine-induced synchronization and is rescued by inducible human wild-type *CEP164* (*p<0.05) but not by human mutant Q525X. After transfection with either control or *Cep164* siRNA cells were released from second thymidine block for 6 hrs. In addition, overexpression of the human truncating mutant *N-GFP-CEP164-Q525X* (dark grey) leads to block in S-phase, indicating a dominant negative effect of the human truncating mutant. P-values were calculated using two-way ANOVA and Bonferroni multiple comparison test.
(TIF)

Figure S3 Expression levels of fibrosis markers in IMCD3 cells. (A–F) Relative gene expression levels of *Tieg1* (A), *TGFβ1* (B), *αSma* (C), *Fibronectin1* (D), *CTGF* (E) and *Cep164 (F)* as measured by RT-QPCR in IMCD3 cells, normalized to *RPL27*. Total RNA was isolated 6 days after transfection with siControl or siCep164-p oligos (A) After 6 days of transient transfection (two rounds) *Tieg1* mRNA levels are significantly (**p<0.01) increased. (B) After 6 days and two rounds of siRNA transfection, *TGFβ1* mRNA levels are significantly (*p<0.05) increased, (C) After 6 days of transient transfection *αSma* mRNA levels are significantly (*p<0.05) increased. (D) After 6 days of transient transfection *Fibronectin1* mRNA levels are significantly (*p<0.05) increased. (E) After 6 days of transient transfection *CTGF* mRNA levels are significantly (*p<0.05) increased. (F) After 6 days of transient transfection *Cep164* mRNA levels are significantly (***p<0.001) decreased (n = 4, error bars represent SEM).
(TIF)

Figure S4 Expression levels of fibrosis markers in MEFs. (A–F) Relative gene expression levels of *Tieg1* (A), *TGFβ1* (B), *αSma* (C), *Fibronectin1* (D), *CTGF* (E) and *Cep164 (F)* as measured by RT-QPCR in MEFs, normalized to *RPL27*. Total RNA was 6 days and after two rounds of siRNA transfection with siControl or siCep164-p oligos (A) After 6 days *Tieg1* mRNA levels are not changed (B) After 6 days of transient transfection *TGFβ1* mRNA levels are significantly (*p<0.05) increased. (C) After 6 days *αSma* mRNA levels are not changed (D) After 6 days of transient transfection *Fibronectin1* mRNA levels are significantly (*p<0.05) increased. (E) After 6 days of transient transfection *CTGF* mRNA levels are significantly (**p<0.01) increased. (F) After 6 days of transient transfection *Cep164* mRNA levels are significantly (*p<0.05) decreased. (n = 3, error bars represent SEM).
(TIF)

Figure S5 Validation of RPE cells expressing *N-GFP- CEP164* alleles and quantification of apoptosis and EMT. (A) Induction of *N-GFP-CEP164* wild-type allele in RPE cells with doxycycline results in expression of GFP-tagged CEP164 at the base of the cilium as shown by immunofluorescence. Centrosomes were stained with pericentrin (magenta) and cilia with acetylated tubulin (white). (B) Functional testing of *CEP164* alleles shows rescue of ciliation after knockdown of endogenous *CEP164* after induction of wild-type human *CEP164* (**p<0.01). Ciliary frequency was quantified from RPE cells with stable *CEP164* constructs transfected with siCtrl (white) or si*CEP164-p* (black) *or siCEP164-i* (patterned) and normalized to siCtrl. Transient knockdown results in significant loss of ciliation (***p<0.001). (>250 cells scored per condition, error bars represent SEM.). P-values were calculated using two-way ANOVA and Bonferroni multiple comparison test. (C) Immunofluorescence imaging of Annexin V (magenta) and Caspase-3 substrate (green) using NucView dual apoptosis assay for live cells. RPE cells were stained 16 hours after knockdown. More apoptosis is observed after knockdown of *CEP164* compared to control. Scale bar represents 50 µm. (D) Five fields per condition were quantified for Caspase-3 and Annexin V, student's t-test was used to calculate difference between siControl of siCEP164-p transfected samples (**p<0.01; n = 3, SEM). (E) Relative gene expression levels of *Snail* as measured by RT-QPCR in RPE cells, normalized to *RPLP0*. Total RNA was isolated 6 days after transfection with siControl, siCEP164-i or siCEP164-p oligos (p = NS, n = 3, SEM), (F) Relative gene expression levels of *Vimentin* as measured by RT-QPCR in RPE cells, normalized to *RPLP0*. Total RNA was isolated 6 days after transfection with siControl, siCEP164-i or siCEP164-p oligos (p = NS, n = 3, SEM), (G) Quantification of absolute distance (µm) of cell migration after 18 hours after a scratch. RPE *CEP164* depleted cells do not migrate more than siControl cells 48 hour after transfection. TGFβ incubation (5 ng/mL) in serum-free medium did not enhance this effect (n = 3, error bars represent SEM). P-values were calculated using two-way ANOVA and Bonferroni multiple comparison test.
(TIF)

Figure S6 Induction of profibrotic gene expression in zebrafish. mRNA expression from 12 pooled embryos is normalized to 2 nL control MO injected zebrafish. (A) RT-QPCR reveals significant induction of *snail* in cep164 MO injected embryos at 72 hpf. (B) RT-QPCR reveals significant induction of *fibronectin1* in cep164 MO injected embryos at 96 hpf. Student's t-test was used to calculate p-values (*<0.05, **<0.01).
(TIF)

Movie S1 Real time imaging of cell cycle progression in control transfected RPE-FUCCI cells. Representative movie of RPE-FUCCI cells transiently transfected with siControl oligos which are imaged for 3 days to examine cell cycle progression. http://syscilia.org/Giles/Supplemental_movie_1_RPE_FUCCI_siControl.mp4.
(MP4)

Movie S2 Real time imaging of cell cycle progression in siCEP164 transfected RPE-FUCCI cells. Representative movie of RPE-FUCCI cells transiently transfected with siCEP164-p oligos which are imaged for 3 days to examine cell cycle progression. http://syscilia.org/Giles/Supplemental_movie_2_RPE_FUCCI_siCEP164.mp4.
(MP4)
