# Supplementary figures and images for: Nephronophthisis-Associated CEP164 Regulates Cell Cycle Progression, Apoptosis and Epithelial-to-Mesenchymal Transition
Source: PLoS Genet. 2014 Oct 23;10(10):e1004594. doi: 10.1371/journal.pgen.1004594 (PMC4207587; doi:10.1371/journal.pgen.1004594)

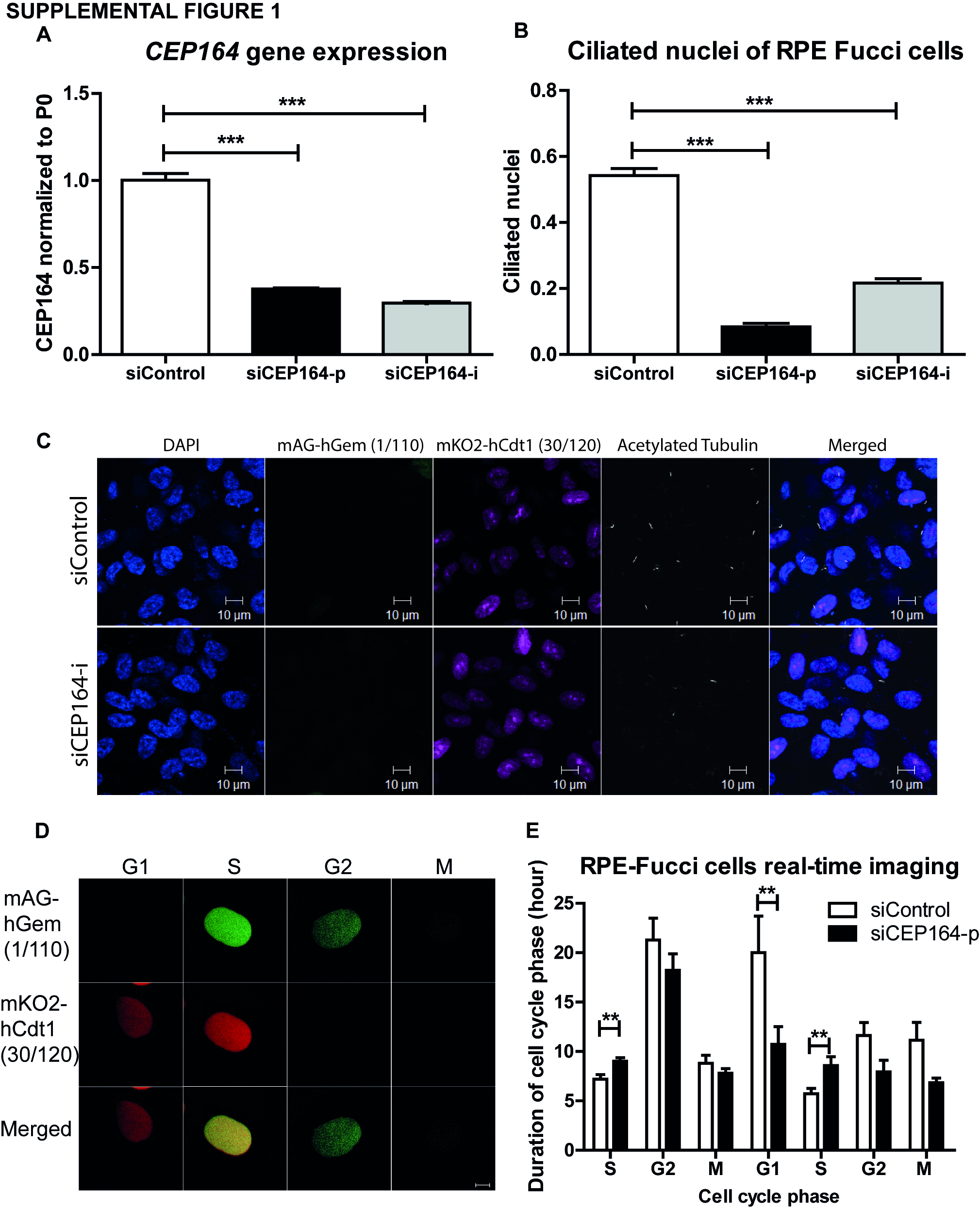

Supplement: Figure S1 — Validation of RPE-FUCCI cells and knockdown of human CEP164. (A) Relative CEP164 gene expression levels as measured by RT-QPCR in RPE-FUCCI cells, normalized to RPLP0. Total RNA was isolated 48 hours after transfection with siControl or siCEP164-p or -i oligos. After 48 hour of transient transfection CEP164 levels are significantly reduced (***p<0.001) (one-way ANOVA (Dunnett's post hoc)) (n = 3, error bars represent SEM). (B) Depletion of CEP164 by siRNA causes a ciliary defect in RPE-FUCCI cells 55 hours after transfection, of which the last 30 hours are serum-starved (***p<0.0001). Nuclei and cilia were scored to generate ciliary frequencies. siCEP164 transfected cells manifest lower cilia frequencies (8–20%) compared to control transfected RPE FUCCI cells (50%). 300 cells per condition were analyzed. Error bars represent SEM. (one-way ANOVA (Dunnett's post hoc)). (C) RPE-FUCCI cells have a primary cilium in G1- and S-phase of the cell cycle, but not during G2- or M-phase (see also Figure 1D). Cells were immunostained for acetylated tubulin (white) and DAPI stains nuclei (blue). Scale bar represents 10 µm. (D) Fluorescence images of RPE-FUCCI cells expressing mKO2-hCdt1(30/120) during G1/early S-phase and mAG-hGem(1/110) constructs during the complete S-phase/G2 phase of the cell cycle, as previously characterized [15]. Cells expressing both construct simultaneously appear yellow/orange, which we classify as early S-phase. Mitotic cells express neither of these constructs and are not fluorescent. Scale bar represents 5 µm. (E) RPE-FUCCI cells and their daughter cells after mitosis are followed during 72 hours after transfection. Duration of each cell cycle stage in siControl and siCEP164 transfected cells was measured. S-phase took significantly longer in siCEP164 transfected cells and their daughter cells compared to control (**p<0.01). G1 phase was significantly shorter in siCEP164 transfected cells compared to control (*p<0.05). G2- (p = 0.06) and M- (p = 0.06 [file pgen.1004594.s001.tif]

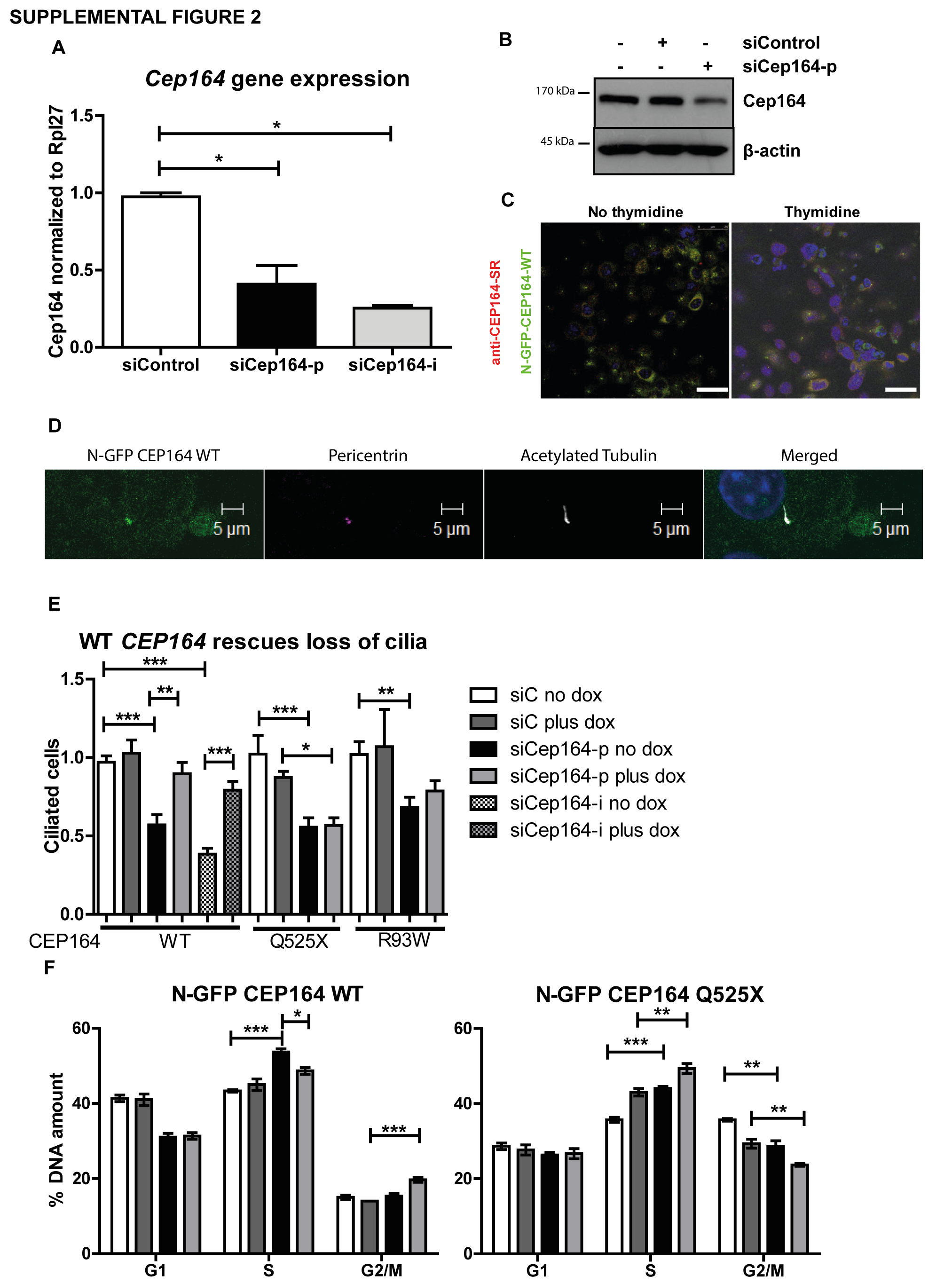

Supplement: Figure S2 — Validation of IMCD3 cells expressing N-GFP- CEP164 alleles and knockdown of mouse Cep164. (A) Cep164 mRNA expression 48 hour after siRNA transfection normalized to RPL27. (*p<0.05) One-way ANOVA, Dunnett's multiple comparisons test (n = 3, error bars represent SEM). (B) Western blot of IMCD3 cells transfected with siCep164-p with β-actin as loading control to quantify protein levels after knockdown. (C) Clonally doxycycline (Dox)-inducible IMCD3 cell line expressing human N-GFP-CEP164-WT was treated with doxycycline (10 ng/mL) during the double thymidine block (2 mM). Cells were fixed and stained with CEP164-SR antibody to observe colocalization. Distinct centrosomal localization of N-GFP-CEP164-WT (green), in the presence or absence of thymidine was observed when costained with CEP164-SR antibody (red). Scale bars represent 25 µm. (D) Induction of N-GFP-CEP164 wild-type allele in IMCD3 cells with doxycycline results in expression of GFP-tagged CEP164 at the base ot he cilium as shown by immunofluorescence. Centrosomes were stained with Pericentrin (magenta) and cilia with Acetylated Tubulin (white). (E) Functional testing of CEP164 alleles shows rescue of ciliation after knockdown of mouse Cep164 after induction of wild-type human CEP164 (**p<0.01) but not mutants (Q525X and R93W). Ciliary frequency was quantified from IMCD3 cells with stable CEP164 constructs transfected with siCtrl (white) or siCep164 (black) and normalized to siCtrl. Transient knockdown results in significant loss of ciliation (***p<0.001). (>250 cells scored per condition, error bars represent SEM.). P-values were calculated using two-way ANOVA and Bonferroni multiple comparison test. (F) Endogenous Cep164 knockdown in a non-clonally selected IMCD3 cell line leads to a block in S-phase under thymidine-induced synchronization and is rescued by inducible human wild-type CEP164 (*p<0.05) but not by human mutant Q525X. After transfection with either control or Cep164 siRNA cells were released from [file pgen.1004594.s002.tif]

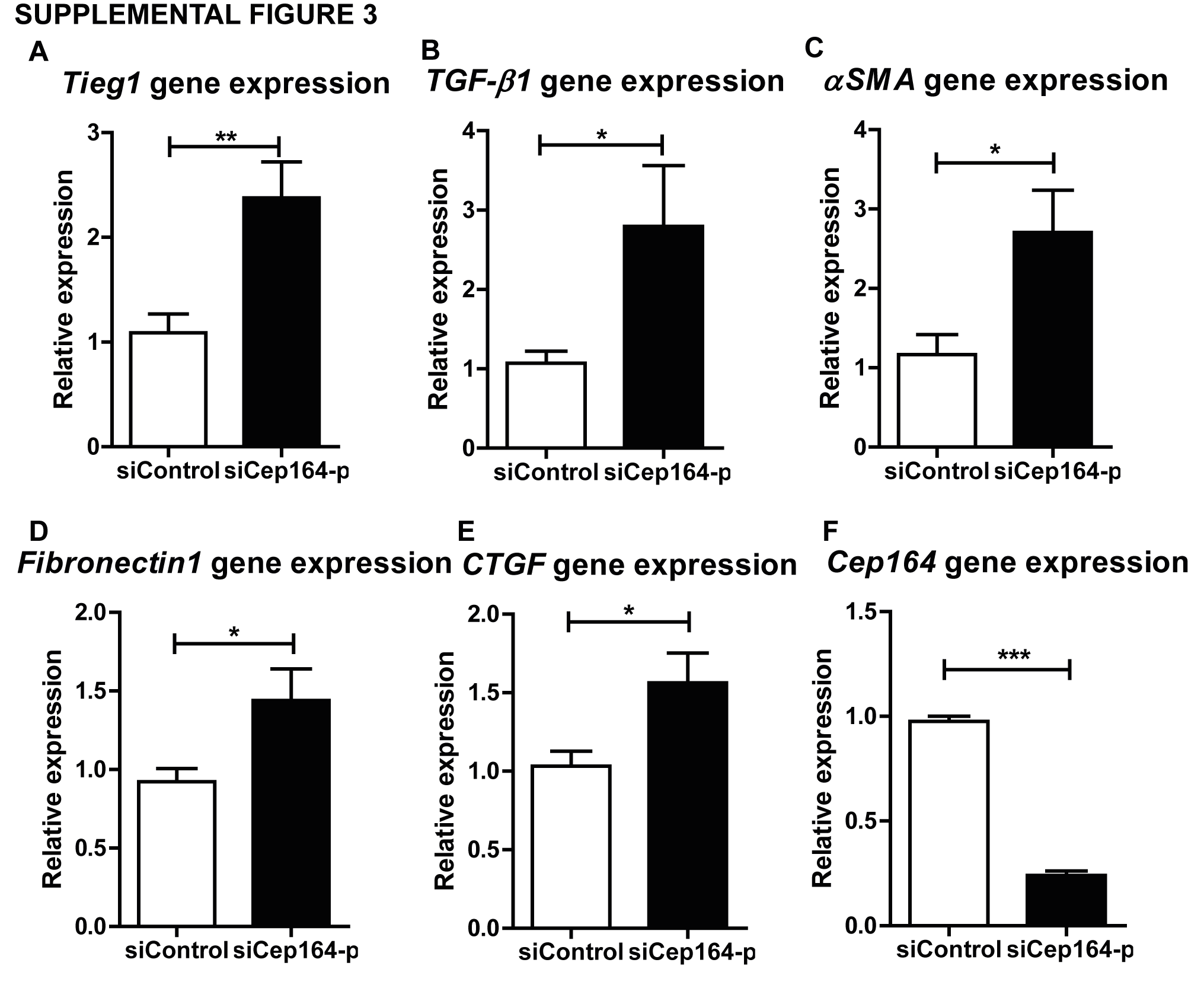

Supplement: Figure S3 — Expression levels of fibrosis markers in IMCD3 cells. (A–F) Relative gene expression levels of Tieg1 (A), TGFβ1 (B), αSma (C), Fibronectin1 (D), CTGF (E) and Cep164 (F) as measured by RT-QPCR in IMCD3 cells, normalized to RPL27. Total RNA was isolated 6 days after transfection with siControl or siCep164-p oligos (A) After 6 days of transient transfection (two rounds) Tieg1 mRNA levels are significantly (**p<0.01) increased. (B) After 6 days and two rounds of siRNA transfection, TGFβ1 mRNA levels are significantly (*p<0.05) increased, (C) After 6 days of transient transfection αSma mRNA levels are significantly (*p<0.05) increased. (D) After 6 days of transient transfection Fibronectin1 mRNA levels are significantly (*p<0.05) increased. (E) After 6 days of transient transfection CTGF mRNA levels are significantly (*p<0.05) increased. (F) After 6 days of transient transfection Cep164 mRNA levels are significantly (***p<0.001) decreased (n = 4, error bars represent SEM). (TIF) [file pgen.1004594.s003.tif]

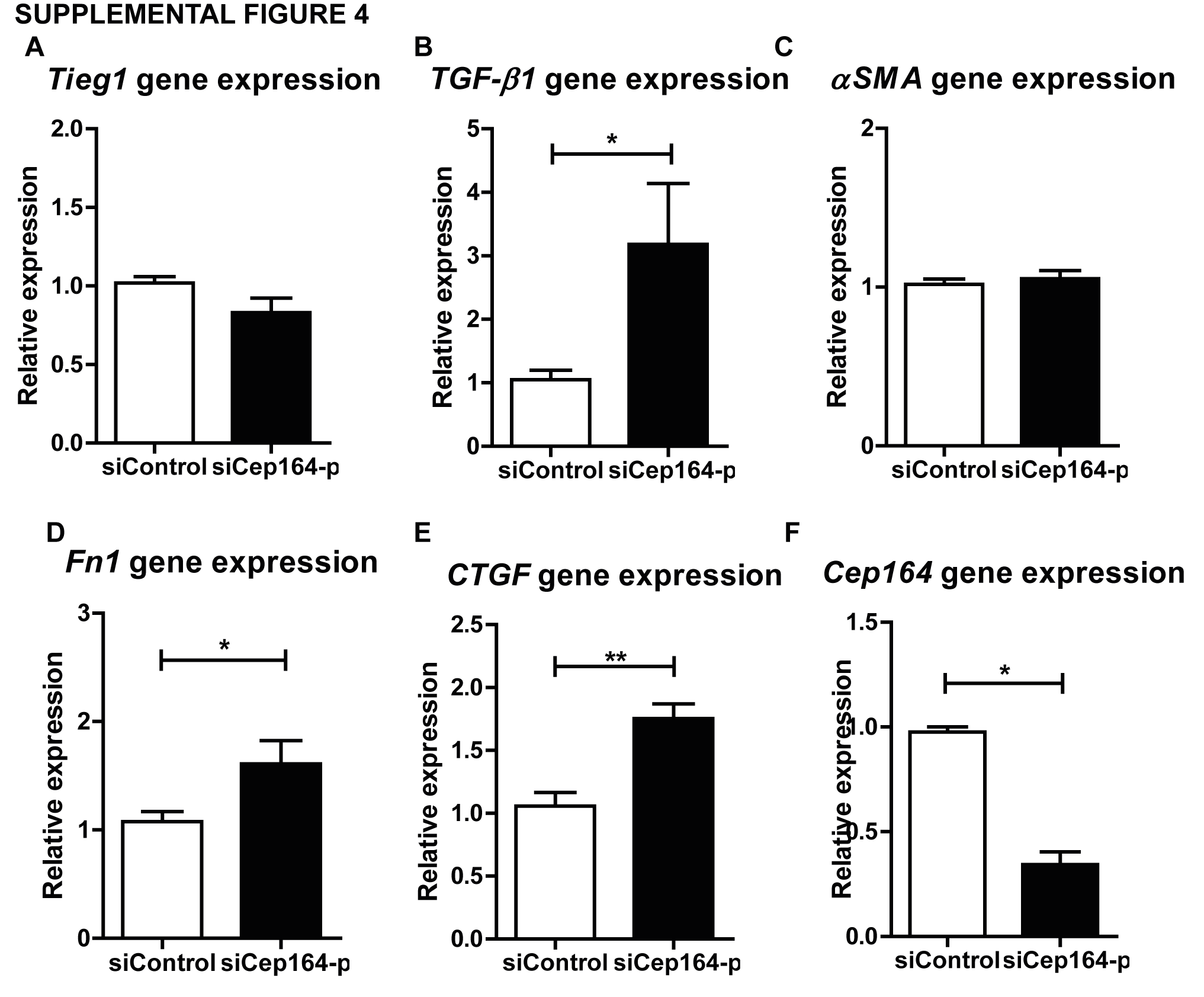

Supplement: Figure S4 — Expression levels of fibrosis markers in MEFs. (A–F) Relative gene expression levels of Tieg1 (A), TGFβ1 (B), αSma (C), Fibronectin1 (D), CTGF (E) and Cep164 (F) as measured by RT-QPCR in MEFs, normalized to RPL27. Total RNA was 6 days and after two rounds of siRNA transfection with siControl or siCep164-p oligos (A) After 6 days Tieg1 mRNA levels are not changed (B) After 6 days of transient transfection TGFβ1 mRNA levels are significantly (*p<0.05) increased. (C) After 6 days αSma mRNA levels are not changed (D) After 6 days of transient transfection Fibronectin1 mRNA levels are significantly (*p<0.05) increased. (E) After 6 days of transient transfection CTGF mRNA levels are significantly (**p<0.01) increased. (F) After 6 days of transient transfection Cep164 mRNA levels are significantly (*p<0.05) decreased. (n = 3, error bars represent SEM). (TIF) [file pgen.1004594.s004.tif]

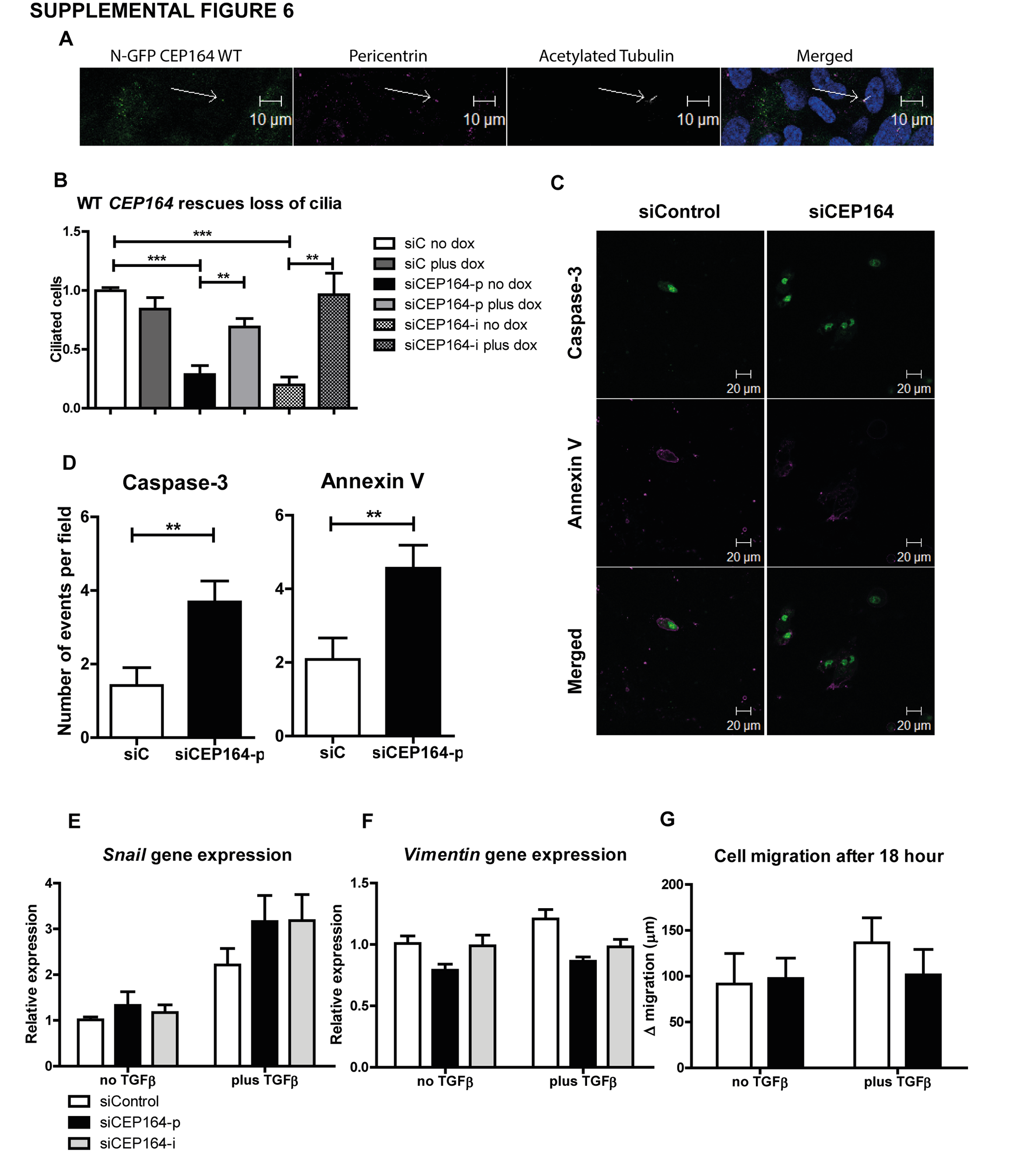

Supplement: Figure S5 — Validation of RPE cells expressing N-GFP- CEP164 alleles and quantification of apoptosis and EMT. (A) Induction of N-GFP-CEP164 wild-type allele in RPE cells with doxycycline results in expression of GFP-tagged CEP164 at the base of the cilium as shown by immunofluorescence. Centrosomes were stained with pericentrin (magenta) and cilia with acetylated tubulin (white). (B) Functional testing of CEP164 alleles shows rescue of ciliation after knockdown of endogenous CEP164 after induction of wild-type human CEP164 (**p<0.01). Ciliary frequency was quantified from RPE cells with stable CEP164 constructs transfected with siCtrl (white) or siCEP164-p (black) or siCEP164-i (patterned) and normalized to siCtrl. Transient knockdown results in significant loss of ciliation (***p<0.001). (>250 cells scored per condition, error bars represent SEM.). P-values were calculated using two-way ANOVA and Bonferroni multiple comparison test. (C) Immunofluorescence imaging of Annexin V (magenta) and Caspase-3 substrate (green) using NucView dual apoptosis assay for live cells. RPE cells were stained 16 hours after knockdown. More apoptosis is observed after knockdown of CEP164 compared to control. Scale bar represents 50 µm. (D) Five fields per condition were quantified for Caspase-3 and Annexin V, student's t-test was used to calculate difference between siControl of siCEP164-p transfected samples (**p<0.01; n = 3, SEM). (E) Relative gene expression levels of Snail as measured by RT-QPCR in RPE cells, normalized to RPLP0. Total RNA was isolated 6 days after transfection with siControl, siCEP164-i or siCEP164-p oligos (p = NS, n = 3, SEM), (F) Relative gene expression levels of Vimentin as measured by RT-QPCR in RPE cells, normalized to RPLP0. Total RNA was isolated 6 days after transfection with siControl, siCEP164-i or siCEP164-p oligos (p = NS, n = 3, SEM), (G) Quantification of absolute distance (µm) of cell migration after 18 hours after a scratch. RPE CEP164 depleted cells do not [file pgen.1004594.s005.tif]

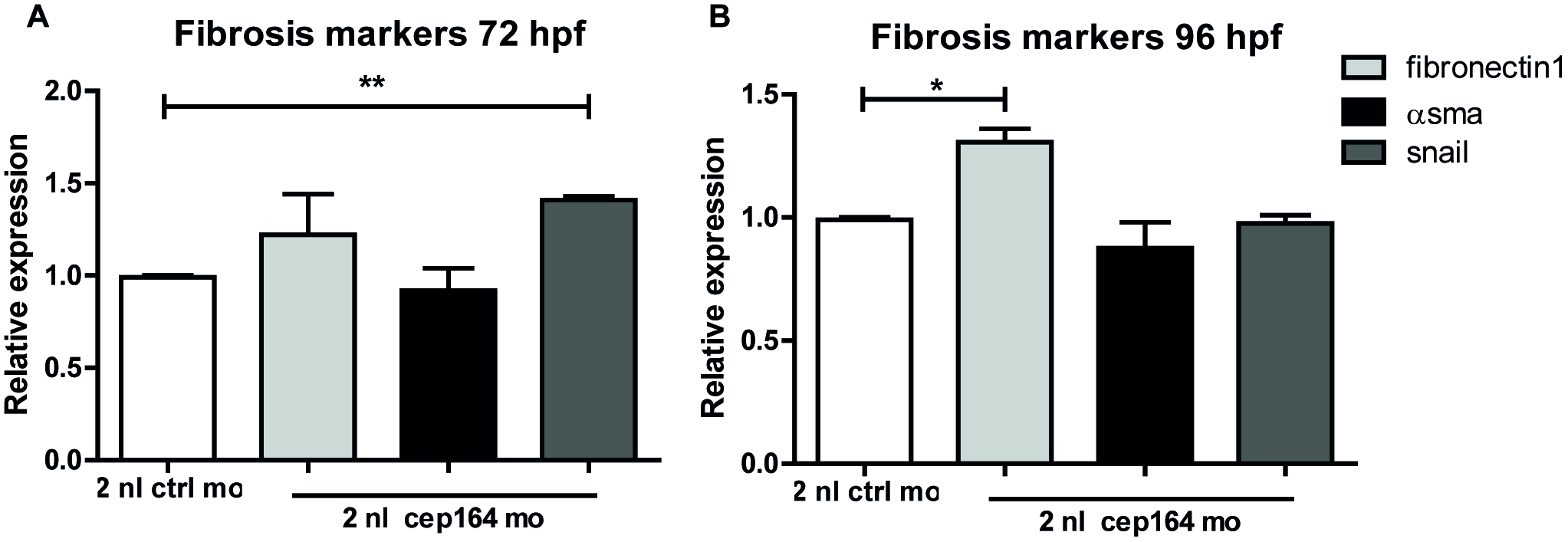

Supplement: Figure S6 — Induction of profibrotic gene expression in zebrafish. mRNA expression from 12 pooled embryos is normalized to 2 nL control MO injected zebrafish. (A) RT-QPCR reveals significant induction of snail in cep164 MO injected embryos at 72 hpf. (B) RT-QPCR reveals significant induction of fibronectin1 in cep164 MO injected embryos at 96 hpf. Student's t-test was used to calculate p-values (*<0.05, **<0.01). (TIF) [file pgen.1004594.s006.tif]
